# Supplementary material for: Maternal vitamin B12, folate during pregnancy and neurocognitive outcomes in young adults of the Pune Maternal Nutrition Study (PMNS) prospective birth cohort: study protocol
Source: BMJ Open. 2021 Sep 22;11(9):e046242. doi: 10.1136/bmjopen-2020-046242 (PMC8461273; doi:10.1136/bmjopen-2020-046242)
Supplement: Supplementary data [file bmjopen-2020-046242supp001.pdf]

	n	Median (Q1, Q3)
**Maternal nutritional variables at 18 wks pregnancy**		
Total calorie intake (kcal)	796	1736.5<br>(1402.4,2074.3)
Plasma B12 (pmol/L)	716	135.1<br>(103.4,175.3)
Red cell folate (ng/mL)	698	388.0<br>(306,490.3)
Plasma Homocysteine (micromol/L)	717	8.2<br>(6.8,10.4)
Serum Ferritin (microgm/L)	745	13.0<br>(8,23)
**Maternal nutritional variables at 28 wks pregnancy**		
Total calorie intake (kcal)	749	1623.8<br>(1318.8,1974.8)
Plasma B12 (pmol/L)	656	121.5<br>(93.5 , 156.9)
Red cell folate (ng/mL)	617	421.0<br>(326,556.5)
Plasma Homocysteine (micromol/L)	653	8.6<br>(6.7,10.9)
Serum Ferritin (microgm/L)	651	11.0<br>(7, 20)

**Birth characteristics**		
Birth weight adjusted to 40 wks (gms)	718	2722.2<br>(2509.8,2948.0)
Length at birth adjusted to 40 wks (cms)	708	48.2<br>(47.0,49.3)
Head circ at birth adjusted to 40 wks	709	33.4<br>(32.5,34.2)

Supplementary material – 1 Maternal characteristics at 18 and 28 week of pregnancy and birth characteristics of the PMNS cohort

Supplementary material - 2.

Details of behavioural assessment of neurocognitive outcomes

a. Neurocognitive assessment:

Standardized battery of neuropsychological tests to assess Intelligence and the domains of executive functions, working memory, and verbal memory.

1. Wechsler's adult intelligence scale (WAIS-IV): Wechsler Adult Intelligence scale (36) (WAIS)-4th edition measures cognitive ability in adults between the ages of 16 years 0 months and 84 years 11 months. The test provides information on the subject's performance on 10 subtests, 4 sub scales i.e. Verbal Comprehension Index scale (VCI), Perceptual Reasoning Index scale (PRI), Working Memory Index scale (WMI) and Processing Speed Index scale (PSI), and gives a final measure of Full-scale IQ (FSIQ).
2. Wechsler's Memory Scale: Wechsler Memory Scale (WMS) (37) measures memory functioning in adults between the ages of 16 years and 90 years. WMS is comprised of eleven sub-tests, and the person's performance is reported on six indices. In this study, six sub-tests will be administered to measure performance on visual immediate memory, visual delayed memory and working memory.
3. Auditory verbal learning test: This is a test of verbal learning and memory (38). The individual is read out a list of 15 words and is asked to recall the words. The list (list A) is initially read out 5 times and recall is tested after each reading. After this, a second list is read out (list B) and recall is tested. Following the recall of list B, recall of list A is immediately tested again. After a 20-minute gap, the delayed recall of list A is tested. The individual is scored based on number of correct answers, omissions, commissions, and total errors.

4. Color trail test: This is a test of executive functions. It involves of 2 parts A & B where subjects have to connect numbered circles from 1 to 25 followed by connecting numbered circles in alternating colors (yellow and pink). Time to complete task and number of errors are recorded. Longer time indicates poor performance.

b. Psychological assessments:

Adult Temperament Questionnaire (ATQ): This 60-item self-report questionnaire is part of Rothbart's scales of temperament. It Yields scores on the domains of negative affect, effortful control, extraversion and orienting sensitivity

c. Mental Health assessment

Brief symptom inventory (BSI) – This is a self or interviewer administered questionnaire that evaluates psychological distress and psychiatric disorders in people. It consists of 53 items covering nine symptom dimensions. Participants rate their feelings about a given item on a 5-point rating scale ranging from 0 (not at all) to 4 (extremely). Rankings characterize the intensity of distress during the past seven days. The test yields score on 9 psychopathology domains and three global indices of distress: Global Severity Index, Positive Symptom Distress Index, and Positive Symptom Total.

MINI (Mini Neuropsychiatric interview plus 7.0): All subjects will be assessed on a structured clinical interview (39) which yields syndromal psychiatric diagnosis based on the DSM-5**.** CMD of interest assessed would be depression and anxiety disorders. All subjects meeting criteria on the MINI will be reassessed by comprehensive clinical mental status examination to confirm the diagnosis. Any subject found to have syndromal psychiatric diagnosis will be offered treatment as per standard clinical protocols.

d. Early life stress: WHO Adverse Childhood Experiences International Questionnaire (ACE-IQ) is a subject rated questionnaire to assess ACE upto age 18 years. Responses are coded

into binary scores which are summated to yield a total ACE-IQ score. A higher score indicates greater ACE.
