# Supplementary material for: Maternal vitamin B12, folate during pregnancy and neurocognitive outcomes in young adults of the Pune Maternal Nutrition Study (PMNS) prospective birth cohort: study protocol
Source: BMJ Open. 2021 Sep 22;11(9):e046242. doi: 10.1136/bmjopen-2020-046242 (PMC8461273; doi:10.1136/bmjopen-2020-046242)
Supplement: Author's manuscript [file bmjopen-2020-046242.draft_revisions.pdf]

BMJ Open is committed to open peer review. As part of this commitment we make the peer review history of every article we publish publicly available.

When an article is published we post the peer reviewers' comments and the authors' responses online. We also post the versions of the paper that were used during peer review. These are the versions that the peer review comments apply to.

The versions of the paper that follow are the versions that were submitted during the peer review process. They are not the versions of record or the final published versions. They should not be cited or distributed as the published version of this manuscript.

BMJ Open is an open access journal and the full, final, typeset and author-corrected version of record of the manuscript is available on our site with no access controls, subscription charges or pay-per-view fees (http://bmjopen.bmj.com).

If you have any questions on BMJ Open's open peer review process please email info.bmjopen@bmj.com

BMJ Open

Developmental origins of neurocognitive outcomes in young adults of the Pune Maternal Nutrition Study (PMNS) prospective birth cohort

Journal:	*BMJ Open*
Manuscript ID	bmjopen-2020-046242
Article Type:	Protocol
Date Submitted by the Author:	28-Oct-2020
Complete List of Authors:	Behere, Rishikesh; KEM Hospital Pune Research Centre, Diabetes Unit<br>Deshpande, Gopikrishna; Auburn University, AU MRI Research Center, Department of Electrical and Computer Engineering, Department of Psychological Sciences, Center for Neuroscience<br>Bandyopadhyay, Souvik; Indian Institute of Public Health<br>Yajnik, Chittaranjan; KEM Hospital Pune, Diabetes Unit, KEMHRC
Keywords:	NUTRITION & DIETETICS, Magnetic resonance imaging < RADIOLOGY & IMAGING, PUBLIC HEALTH

SCHOLARONE™
Manuscripts

I, the Submitting Author has the right to grant and does grant on behalf of all authors of the Work (as defined in the below author licence), an exclusive licence and/or a non-exclusive licence for contributions from authors who are: i) UK Crown employees; ii) where BMJ has agreed a CC-BY licence shall apply, and/or iii) in accordance with the terms applicable for US Federal Government officers or employees acting as part of their official duties; on a worldwide, perpetual, irrevocable, royalty-free basis to BMJ Publishing Group Ltd ("BMJ") its licensees and where the relevant Journal is co-owned by BMJ to the co-owners of the Journal, to publish the Work in this journal and any other BMJ products and to exploit all rights, as set out in our licence.

The Submitting Author accepts and understands that any supply made under these terms is made by BMJ to the Submitting Author unless you are acting as an employee on behalf of your employer or a postgraduate student of an affiliated institution which is paying any applicable article publishing charge ("APC") for Open Access articles. Where the Submitting Author wishes to make the Work available on an Open Access basis (and intends to pay the relevant APC), the terms of reuse of such Open Access shall be governed by a Creative Commons licence – details of these licences and which Creative Commons licence will apply to this Work are set out in our licence referred to above.

Other than as permitted in any relevant BMJ Author's Self Archiving Policies, I confirm this Work has not been accepted for publication elsewhere, is not being considered for publication elsewhere and does not duplicate material already published. I confirm all authors consent to publication of this Work and authorise the granting of this licence.

For peer review only

Developmental origins of neurocognitive outcomes in young adults of the Pune Maternal Nutrition Study (PMNS) prospective birth cohort

Rishikesh V. Behere[1*], Gopikrishna Desphande[2], Souvik Bandyopadhyay[3], Chittaranjan S Yajnik[4]

1- India Alliance Intermediate Fellow, KEM Hospital Research Center, Pune, India

2 - Professor, AU MRI Research Center, Department of Electrical and Computer Engineering, Department of Psychological Sciences, Center for Neuroscience, Auburn University, Alabama, USA; Alabama Advanced Imaging Consortium, Birmingham, Alabama, USA

3 – Biostatistician and program manager, GSK Asia. (Work was done as Assistant Professor, Indian Institute of Public Health, Public Health Foundation of India, Hyderabad, India)

4 - Director, Diabetes Unit, KEM Hospital Research Center, India

[*] **Corresponding Author**

Dr. Rishikesh V. Behere

India Alliance Intermediate Fellow

Diabetes Unit

KEM Hospital Research Center

Pune, India

Email: rvbehere@gmail.com

Ph no: +912026061958

For peer review only

Abstract:

Introduction:

The Developmental origins of Health and Disease (DOHaD) hypothesis, proposes that intrauterine and early life exposures significantly influence fetal development and risk for disease in later life. Evidence from prospective birth cohorts suggests a role for maternal B12 and folate in influencing neurocognitive outcomes in the offspring. In the Indian setting, B12 deficiency is common during pregnancy while rates of folate deficiency are lower. The long term influences of maternal nutrition during pregnancy on adult neurocognitive outcomes have not been examined. The Pune Maternal Nutrition Study (PMNS) is a pre-conceptional birth cohort into its 24th year and is considered a unique resource to study the DOHaD hypothesis. We found an association between maternal B12 status in pregnancy and child's neurocognitive status at 9 years of age. We now plan to assess neurocognitive function and MRI measurements of brain structural-functional connectivity at young adult age to study its association with maternal nutritional exposures during pregnancy.

Methods and analysis:

As part of ongoing prospective follow up in young adults of the PMNS at the Diabetes Unit, KEM Hospital Research Center, Pune India, the following measurements will be done: Neurocognitive performance (Standardized tests of Intelligence, verbal and visual memory, attention and executive functions), temperament (Adult temperament questionnaire), psychopathology (Brief symptom inventory and clinical interview on Mini Neuropsychiatric Interview 7.0). Brain Magnetic Resonance Imaging (MRI) for structural T1, resting-state functional connectivity and diffusion tensor imaging will be performed on a subset of the cohort (selected based on exposure to a lower or higher maternal B12 status at 18 weeks of pregnancy).

For peer review only

Ethics and dissemination:

The study is approved by Institutional ethics committee of KEM Hospital Research Center, Pune. The results will be shared at national and international scientific conferences and published in peer reviewed scientific journals.

Trial Registration: ClinicalTrials.gov ID: NCT03096028

Keywords: maternal nutrition, vitamin B12, neurocognitive development, brain MRI, birth cohort,

Strengths and Limitations:

- The Pune Maternal Nutrition Study (PMNS) is a preconceptional birth cohort in the 24th year of its follow up and is considered a unique resource to study the DOHaD paradigm.
- The study will measure neurocognitive and mental health outcomes in young adult offspring of the PMNS and utilise serial life course information on the cohort to examine factors influencing neurodevelopment.
- We propose to use a multi-modal brain imaging approach (structural and functional connectivity) to provide greater insights into the mechanistic effects of early life exposures on long term neurocognitive outcomes.
- Neuroimaging proposed is cross-sectional, and we do not have longitudinal imaging data from younger years of the cohort, which would have allowed us to map developmental trajectories.

For peer review only

Introduction:

The Developmental origins of Health and Disease (DOHaD) hypothesis(1,2) proposes that early life exposures (intrauterine environment including maternal nutritional and metabolic factors) during critical periods of development affect structure and function of developing organs. This could in turn influence the health and risk for chronic disease in later life (programming). Over the life course, the risk may be modified by post-natal environmental exposures (childhood experiences, education, nutrition, infections, lifestyle, socio-economic conditions). Recent investigations suggest an early life origin of neurodevelopmental disorders (autism spectrum disorders, depression, schizophrenia) (3). Disturbed temperamental traits, poorer neurocognitive functions and altered brain structural-functional connectivity are risk markers for these disorders (4–7).

Maternal nutritional status is an important factor influencing early foetal growth and development. A systematic review reported associations of low micronutrient status with poorer offspring cognitive functions(8). Deficiency of various micronutrients during critical phases of foetal development can affect organ structure and function, hence programming the risk for chronic disease in later life(9).

Observations from the follow up of children born to mothers who faced severe calorie restriction at the time of pregnancy during the Dutch winter hunger(10) and Chinese famine(11) support the DOHaD hypothesis. The offspring had lower cognitive functions and a two times higher risk for schizophrenia and depression in adulthood than the general population(12–14). However, these observations may be influenced by several confounding factors (maternal stress, offspring nutrition, growth and development, environmental exposures etc). Prospective birth cohorts with systematic examination of maternal health, nutrition, serial foetal growth and development, birth outcomes and subsequent childhood

For peer review only

growth and development, as well as environmental exposures provide a better understanding of the life course model affecting the risk for later life neurocognitive disorders(15).

Recently there is considerable interest in the maternal concentrations of micronutrients vitamin B12 and folate, both of which influence the one carbon metabolism cycle(16). These micronutrients are important for purine pyrimidine synthesis, DNA repair, and DNA methylation reactions that can influence genetic expression (epigenetic processes). Maternal B12 folate deficiency and hyperhomocysteinemia during pregnancy have been described to be associated with neural tube defects, low birth weight, small for gestational age and developmental delay(17–19). Animal models have examined the role of a maternal B12-folate micronutrient imbalance and show that offspring dams of mothers fed on a lowB12-highfolate diet have poorer spatial memory functions, lower brain hippocampal weight, reduced neurotrophic factor BDNF and reduced TRK gene expression(20,21). Observations from large prospective cohort studies such as the Norwegian mother and child birth cohort (MoBa)(22) and Generation R support association of poor maternal folate status with poorer neurocognitive functioning, greater childhood behavioral problems(23–25), smaller brain volumes(26) and risk for autism(27,28). The Avon's Longitudinal Study on Parents And Children (ALSPAC) reported a weak causality for association between low maternal dietary intake of vitamin B12 and lower IQ scores at 8 years by Mendelian randomization analysis using genetic determinants of B12 (FUT2gene) (29).

In the Indian setting, vitamin B12 deficiency is widely prevalent in women in the reproductive age group while rates of folate deficiency are low (30–32). Public health policy mandates only iron-folic acid supplementation in adolescents and pregnant women. It is also a common clinical practice to supplement high dose folic acid (5mg) to pregnant women. This promotes an imbalance between vitamin B12 and folate status during pregnancy(33) which could enhance risk of adverse health outcomes in the offspring

For peer review only

including aberrant neurodevelopment. However, there are few studies in general and more specifically in India examining long term effects of maternal nutrition and developmental factors over the life course on neurodevelopmental outcomes in young adult offspring.

The Pune Maternal Nutrition Study (PMNS) is a pre-conceptional birth cohort with serial data on maternal nutritional status, birth outcomes and offspring nutrition, physical growth and development. The low B12-high folate dietary pattern was associated with adverse metabolic outcomes in the offspring at 6 years[24]. We have earlier demonstrated in this cohort that exposure to a low maternal B12 was associated with poor cognitive functioning in the offspring at 9 years of age(34). The offspring of this cohort are now in their early 20's and this provides a unique opportunity to examine the long-term effects of maternal nutrition status during pregnancy on neurocognitive outcomes in young adulthood.

Aims and objectives:

The key aim of the study is to examine whether maternal micronutrient status during pregnancy (B12, folate) is associated with neurocognitive performance, structural and functional connectivity and risk for common mental disorders in young adult offspring of the PMNS birth cohort.

Primary objectives:

1. To examine associations between maternal vitamin B12, folate, and their interaction at 18 and 28 weeks of pregnancy with neurocognitive outcomes (neuropsychological test performance) and risk for common mental disorders (temperament, presence of depression or anxiety disorders) in young adulthood; controlling for the effects of other nutrients (calorie, protein and fat intake, and levels of vitamin C,D, ferritin) and

For peer review only

later life developmental influences (education, adverse childhood experiences, offspring nutrition, physical growth and development, metabolic status).

2. To examine the association of maternal nutritional status during pregnancy with brain structure (grey matter volume and cortical thickness), and structural/functional connectivity (using Diffusion Tensor Imaging – DTI and resting state functional Magnetic Resonance Imaging – rsfMRI, respectively).
3. To test the causality of association between maternal vitamin B12, folate, and neurocognitive outcomes.

For peer review only

Hypotheses:

We hypothesise that a lower maternal B12, higher folate, higher homocysteine nutrient pattern during pregnancy adversely impacts development of brain regions, as well as structural/functional networks (inclusive of hippocampal, frontal-subcortical connections) involved in cognitive processing (memory, executive functions) and this reflects in poorer neurodevelopmental outcomes (lower neuropsychological test performance, adverse temperamental traits and higher risk for common mental disorders) in the young adult offspring.

Methods and Analysis:

Study setting:

Pune Maternal Nutrition Study:

The PMNS is a preconceptional cohort, established in 6 villages of Pune district, Maharashtra state, India in 1993 in collaboration with Dr DJP Barker and Dr Caroline Fall and is considered a unique resource in the world to examine the DOHaD paradigm. Initially, 2466 women (F0 generation) in the reproductive age group participated in the study. 814

women who became pregnant in the period June 1994 to April 1996 were enrolled for the study. Of these 797 pregnancies (with singleton pregnancy and period of gestation less than 21 weeks) were included. 762 live births occurred (F1 generation). This cohort has data on pre-conceptional parental size, maternal nutrition and size at birth of the offspring. Children are being regularly followed up for growth and development (body size and DXA body composition), nutrition, and cardio-metabolic risk factors (6,12&18 years of age). Biological samples have been collected and stored in a bio-repository and are available for assessments. The other strengths of this cohort are good community participation and follow up rates of over 90% over the years. At 18 years follow up, 690 of the original 762 live births in the cohort were followed up.

The participants (F1 generation) of the cohort are also part of a randomized controlled trial (Pune Rural Intervention in Young Adolescents, PRIYA) (35) to examine intergenerational effects of micronutrient supplementation on various health outcomes. In 2012 at the age of 17 years 557 eligible members were randomized as part of the nutrition intervention trial into one of 3 groups 1) 2 micrograms/day of vitamin B12, 2) 2 micrograms/day of Vitamin B12 with UNIMAPP multivitamins and 20g milk powder 3) placebo**.** The trial was completed in the boys in December 2017 and in the girls in September 2019.

Subject selection:

All subjects of the PMNS cohort (n=690) will be invited for participation in the study (Figure 1). Neurocognitive assessments, temperament and mental health assessments will be performed in all consenting subjects. Brain MRI will be performed on a subset of the cohort selected based on exposure to maternal B12 levels at 18 weeks of gestation. Median B12 level at 18-week gestation was 153pM (103,175 pM being the 1st and 4th quartiles). Description of maternal characteristics at 18 and 28-week gestation and offspring birth characteristics are provided in the supplementary file. Of the 690 participants still in follow

For peer review only

up, 152 subjects (78 males, 74 females) whose mothers had plasma vitamin B12 concentration <103pM and 154 subjects (88 males, 66 females) whose mothers had plasma vitamin B12 concentration >175pM will be eligible for participation in the brain MRI scans. 200 participants from each of these two groups (100 in each group) will be randomly selected.

Assessments:

The following measurements will be performed (Table – 1):

Table -1 Outcomes and exposures defined in the study

	**Outcomes**	**Tests**	**Variables measured**
1.	Neurocognitive performance	WAIS-IV<br>WMS<br>AVLT<br>Color Trails test	Verbal comprehension, perceptual reasoning, working memory, processing speed, Full scale IQ.<br>Immediate Memory, delayed memory and working memory.<br>Errors in Immediate and delayed recall<br>Time taken and errors for Color trails test A & B.
2.	Temperament	Adult Temperament Questionnaire	Negative affect, extraversion, orienting sensitivity, effortful control
3.	Common mental disorders	Brief Symptom Inventory<br>Mini Neuropsychiatric Interview 7.0 for DSM 5 diagnosis	Global severity index<br>DSM5 diagnosis of past or current Major Depressive disorder, suicidality, Panic disorder, Agoraphobia, Social anxiety disorder, generalised anxiety disorder
5.	Brain Imaging	Structural T1 MPRAGE<br>Resting state fMRI<br>DTI	Cortical volume and thickness<br>Resting state functional connectivity<br>Tractography, FA and MD values in relevant white matter tracts
	**Exposures**	**Tests**	**Variables measured**
	Micronutrient	Plasma levels	Vit B12, red cell folate, Ferritin, Vit C

For peer review only

	status and homocysteine at 18 and 28 wks pregnancy		homocysteine
	Nutritional intake	Food frequency questionnaire	Total calorie intake, Protein and fat intake

a. Neurocognitive assessment:

A Standardized battery of neuropsychological tests are administered to assess Intelligence and the domains of executive functions, working memory, and verbal memory.

1. Wechsler's adult intelligence scale (WAIS-IV): Wechsler Adult Intelligence scale (36) (WAIS)-4th edition measures cognitive ability in adults between the ages of 16 years 0 months and 84 years 11 months. The test provides information on the subject's performance on 10 subtests, 4 sub scales i.e. Verbal Comprehension Index scale (VCI), Perceptual Reasoning Index scale (PRI), Working Memory Index scale (WMI) and Processing Speed Index scale (PSI), and gives a final measure of Full-scale IQ (FSIQ).
2. Wechsler's Memory Scale: Wechsler Memory Scale (WMS) (37) measures memory functioning in adults between the ages of 16 years and 90 years. WMS is comprised of eleven sub-tests, and the person's performance is reported on six indices. In this study, six sub-tests will be administered to measure performance on visual immediate memory, visual delayed memory and working memory.
3. Auditory verbal learning test: This is a test of verbal learning and memory (38). The individual is read out a list of 15 words and is asked to recall the words. The list (list A) is initially read out 5 times and recall is tested after each reading. After this, a second list is read out (list B) and recall is tested. Following the recall of list B, recall

For peer review only

of list A is immediately tested again. After a 20-minute gap, the delayed recall of list A is tested. The individual is scored based on number of correct answers, omissions, commissions, and total errors.

4. Color trail test: This is a test of executive functions. It involves of 2 parts A & B where subjects have to connect numbered circles from 1 to 25 followed by connecting numbered circles in alternating colors (yellow and pink). Time to complete task and number of errors are recorded. Longer time indicates poor performance.

b. Psychological assessments:

Adult Temperament Questionnaire (ATQ): This 60-item self-report questionnaire is part of Rothbart's scales of temperament. It Yields scores on the domains of negative affect, effortful control, extraversion and orienting sensitivity

c. Mental Health assessment

Brief symptom inventory (BSI) – This is a self or interviewer administered questionnaire that evaluates psychological distress and psychiatric disorders in people. It consists of 53 items covering nine symptom dimensions. Participants rate their feelings about a given item on a 5-point rating scale ranging from 0 (not at all) to 4 (extremely). Rankings characterize the intensity of distress during the past seven days. The test yields score on 9 psychopathology domains and three global indices of distress: Global Severity Index, Positive Symptom Distress Index, and Positive Symptom Total.

MINI (Mini Neuropsychiatric interview plus 7.0): All subjects will be assessed on a structured clinical interview (39) which yields syndromal psychiatric diagnosis based on the DSM-5**.** CMD of interest assessed would be depression and anxiety disorders. All subjects meeting criteria on the MINI will be reassessed by comprehensive clinical mental status examination to confirm the diagnosis. Any subject found to have syndromal psychiatric diagnosis will be offered treatment as per standard clinical protocols.

For peer review only

d. Early life stress: WHO Adverse Childhood Experiences International Questionnaire (ACE-IQ) is a subject rated questionnaire to assess ACE upto age 18 years. Responses are coded into binary scores which are summated to yield a total ACE-IQ score. A higher score indicates greater ACE.

e. Magnetic resonance imaging

Anatomical data using structural 3D T1 weighted MPRAGE (magnetization prepared rapid gradient echo) sequence, resting state fMRI (rs-fmri) with 2D gradient-echo EPI (echo planar imaging) sequence, and Diffusion Tensor Imaging (DTI) using RESOLVE sequence will be acquired on a 3 Tesla Siemens Skyra scanner.

Image acquisition

For structural imaging, 3D T1-weighted MPRAGE images will be acquired (TR=2300 ms, TE=2.26 ms, TI=900 ms, Flip angle=8 degrees, slice thickness=1 mm, in-plane resolution= 1×1 mm^2, GRAPPA parallel imaging with acceleration factor of 2, 192 slices, FOV readout=256 mm, FOV phase encoding= 100%, and acquisition time of 5 min and 21 s).

For functional imaging, T2*-weighted echo-planar imaging sequence will be used to acquire BOLD fMRI data while participants are resting with eyes open and fixated, and not thinking about anything specific. Thirty six axial slices will be acquired covering the whole brain with a slice thickness of 3 mm, in-plane resolution of 3×3 mm^2, TR=2000 ms; TE=30 ms; flip angle=90^0, FOV readout=256 mm, FOV phase encoding= 100%, GRAPPA parallel imaging with acceleration factor of 2, and 300 measurements with an acquisition time of 10 min and 8 s. PACE (prospective acquisition correction) motion correction will be employed in this sequence. (40,41)

For peer review only

Diffusion-weighted images will be acquired using the RESOLVE (readout segmentation of long variable echo-trains) sequence given its robustness against susceptibility artifacts. Scan parameters for RESOLVE will be: Thirty-six axial slices covering the whole brain with a slice thickness of 3 mm, in-plane resolution of 1.4×1.4 mm^2, TR=5460 ms; TE=73/109 ms; flip angle=180^0, FOV readout=220 mm, FOV phase encoding= 100%, GRAPPA parallel imaging with acceleration factor of 2, 30 diffusion directions, two diffusion weightings (b-values) of 0 and 1000 s/mm^2 and an acquisition time of 14 min and 19 s.

All MRI images will be visually inspected for quality assessment and reviewed by a radiologist to rule out any structural abnormalities.

For pe

Data management:

Data will be stored at the Diabetes Unit, KEM Hospital Research Center, Pune, India. Data collected will be first entered in a paper clinical record form and double entered into an open clinical platform on a computer. Data is downloaded and checked for extreme or missing values. Data on maternal micronutrient status during pregnancy (nutrition intake, circulating micronutrients) and intermediate variables (birth size, childhood nutrition, growth and development, metabolic factors (insulin resistance, glycemic status, adiposity), socio demographic variables and socio-economic status have been assessed previously on the cohort. These will be extracted and used for analysis.

Statistical methods:

Power estimation:

There are 690 members of the cohort available for assessments. Assuming a 80%

participation rate (from experience of previous studies on the cohort and accounting for migration) we estimate that 550 subjects would be recruited for the study. Using a test at 5% significance level, the study will have 80% power to detect an association of 0.11 standard deviation of continuous outcome (eg. neurocognitive test score) per standard deviation of continuous exposure (eg. maternal vitaminB12 level). Study of 200 subjects (100 in each group) for Brain imaging will have 80% power for an effect size 0.3 and significance at p <0.05 for detecting difference between the two maternal B12 exposure groups.

Plan of Data analysis:

Objective 1: This examines the association between intrauterine nutritional exposures and neurocognitive outcomes in young adult age:

An exploratory analysis approach will be employed at first. Distribution of outcome variables such as neurocognitive performance scores will be visualized by using different plotting techniques such as box plot and scatter plot . Univariate analysis (Pearson' correlation and linear regression) will be performed to examine the relationships between maternal nutritional exposures (B12, folate, homocysteine, calorie, protein and fat intake, and levels of vitamin C,D, ferritin) and neurocognitive outcomes (neuropsychological test scores, temperament, presence of depression or anxiety disorders). We will examine our hypothesis of specific associations of maternal B12 and folate with neurocognitive outcomes by constructing multivariate linear (if outcome is continuous) or logistic regression (categorical outcome) models. The models will adjust for covariates (age, gender, education, socio-economic status) and other maternal nutritional exposures. We will extend the models to adjust for variables having potential modifying and mediating effect on the associations (birth size, physical growth, offspring nutrition, parental education, adverse childhood experiences). Finally we will adjust for the B12 intervention status (B12 intervention received yes or no) in the PRIYA trial.

For peer review only

Given the multitude of factors and possible collinearity we will use Principal Component Analysis (PCA) and cluster analysis to achieve dimensionality reduction. The clusters might be used to construct path analytical models for exploring associations between outcomes.

Objective 2: This examines the association between maternal nutritional exposures and brain structural functional connectivity:

1. Structural Brain MRI analysis
    a. Voxel-based Morphometry on T1 MPRAGE anatomical images will be performed using statistical parametric mapping in Matlab. Pre-processing steps will involve co-registration, segmentation. Spatial normalisation will transform all subject data to the same stereotactic space. Gray matter images will be smoothed by convolving with an isotropic Gaussian kernel. Statistical analysis using General Linear Model will be used to identify regions of grey matter concentration that are significantly associated with our exposures of interest.
    b. Cortical volume, thickness and hippocampal segmentation: A surface based morphometric technique will be employed to measure cortical volume and thickness. We will use freesurfer software for image analysis. Standard steps include intensity normalisation, skull stripping and removal of non-brain tissues followed by segmentation, topology correction, registration and automated parcellation. Cortical volume and thickness will be computed for 68 regions (34 in each hemisphere) using the Desikan-Kelliany Atlas. The hippocampal subfield segmentation tool in freesurfer will be employed to compute volumes of the various hippocampal structures as per the inbuilt atlas which defines 11 hippocampal subfield structures.

For peer review only

2. Structural Connectivity – Diffusion tractography analysis: Diffusion weighted images will be analysed using the "The Diffusion Toolbox" software tool in the FMRIB Software Library (FSL; http://www.fmrib.ox.ac.uk/fsl/fdt/index.html). After pre-processing including motion correction and eddy current correction, the DTI indices fractional anisotropy (FA), mean diffusivity (MD) and radial diffusivity (RD) will be computed. Structural connectome generation will be performed on the MRItrix3 software. Pre-processing steps will involve denoising and correction for eddy currents. The b0 volume of pre-processed images will be extracted and registered to the T1 image using FSL's BET tool. The fiber orientation dispersion functions (FODs) will be generated for white matter and normalised. Probabilistic streamlines tractography will be performed by dynamically seeding the normalized white matter FOD's. The whole brain tractogram thus generated will be used for structural connectome generation.

3. Functional Connectivity: CONN software (https://web.conn-toolbox.org/) will be used for the analysis of rs-fMRI data. This will involve standard pre-processing steps such as realignment, normalization to MNI space, elimination of temporal linear trends and regressing out nuisance covariates such as residual motion and physiological artifacts. *aCompCor* will be used for minimizing physiological artifacts. Motion censoring and interpolation will also be used to remove the effects of large frame wise displacements due to head motion. Following pre-processing, mean time series will be extracted from 91 cortical and 15 sub-cortical regions in the FSL Harvard-Oxford atlas, as well as from 26 cerebellar regions in the AAL atlas. These time series will be used in a whole brain functional connectivity analysis employing Pearson's correlation coefficient between the mean time series.

For peer review only

For each modality, the following statistical comparisons will be performed. We will first perform correlations between maternal B12 and folate exposures with brain MRI outcomes to examine for any linear relationships. We will use an analysis of covariance (ANCOVA) model to examine differences in MRI outcomes between low and high maternal B12 groups. Similarly, we will examine for differences between low and high maternal red cell folate groups (selected based on median value of maternal red cell folate) and for any interaction between maternal B12 and folate status. Variance due to subjects' age, gender, education, total intracranial volume, adverse childhood experiences, socio-economic status, current B12 status and maternal education will be regressed out of the analyses.

For making inferences from multimodal imaging data, we will adopt a hierarchical approach. Accordingly, we will perform voxel-wise tests for difference in grey matter volume (or cortical thickness). For brain regions which are significant, DTI-based structural connectivity between those regions will be investigated. For regions whose structural connectivity is also significantly different for the contrasts of interest, fMRI-based functional connectivity between such regions will be tested. This strategy assures that the effects of interest have both structural and functional etiologies, and hence are likely to be robust and reproducible.

Objective 3:

This examines causality of association between maternal nutritional factors and neurocognitive outcomes. Mendelian randomization analysis (triangulation and instrumental variable analysis) using genetic determinant of vitaminB12 (TCN2rs1801198, FUT2rs492602) and homocysteine (MTHFRrs1801133) will be performed.

A model to understand neurocognitive development using a life course approach in depicted in figure 2.

For peer review only

Patient and public involvement:

The present study is a follow up of participants of the Pune Maternal Nutrition Study birth cohort. This cohort was set up in 1993 from six villages around Pune district in India. The cohort has been serially evaluated every 6 years (at 6,12,18 years) and is currently in the 24th year of its follow up. The cohort participants were also part of randomized controlled trial from 2012-2019. We are also following up the children born in the next generation of the cohort. The cohort has a high follow up rate of 90% over the last 18 years. This was possible due to active community engagement and involvement of social workers who are from the same community and interact with cohort participants at regularly. Information on results of our studies are shared with cohort participants through public engagement initiatives.

Ethics and dissemination:

The study will be carried out as per the ethical guidelines provided by the Indian Council of Medical Research. The study has been approved by the Institutional Ethics Committee of the KEM Hospital Research Center, Pune (KEMHRC/RVM/EC/1249). Study is registered with clinicaltrials.gov (ID-NCT 03096028). The cohort participants will be approached by the field workers who have been following them in the community for last many years. Participants will be provided the study information sheet and an opportunity to discuss their doubts and concerns regarding the study with the investigator and then recruited after they sign a written informed consent.

The data generated from this study will be shared with the scientific community at local, national and international conferences, and published in open access peer reviewed journals. The major findings will be shared with the general public, study participants and their families in lay terms through public engagement initiatives.

For peer review only

Conclusion:

The DOHaD hypothesis proposes than intrauterine and early life exposures significantly influence neurodevelopment and enhance risk for common mental disorders in later life. Evidence from prospective birth cohorts suggests a significant role for maternal B12 and folate in influencing neurocognitive (neuropsychological performance) and mental health outcomes (increased risk for autism, ADHD, schizophrenia) in the offspring. In the Indian setting, B12 deficiency is widely prevalent, however public health policy mandates only iron and folic acid supplementation in the reproductive age group. The pre-conceptional birth cohort PMNS is now in its 24th year and provides an unique opportunity to study the effect of maternal nutrition during pregnancy on long term neurocognitive outcomes and risk of common mental disorders in adulthood. The strengths of the cohort are systematic data on serial assessments on the cohort and good community participation and low attrition rates. The study will help build a database on neurocognitive and mental health outcomes and brain imaging in young adulthood in a pre-conceptional birth cohort. The results of the association of maternal B12 during pregnancy and long term neurodevelopmental outcomes will inform public health actions for nutritional health of adolescent girls and women of reproductive age.

Figure 1 - Selection of subjects from the PMNS cohort for the study

Figure 2 – Understanding neurocognitive development using a life course approach

For peer review only

Competing interests:

The authors declare that they have no competing interests.

Funding:

This work is supported by the DBT/Wellcome Trust India Alliance Fellowship [IA/CPHI/161502665] awarded to Dr Rishikesh V. Behere.

Author's contribution:

RVB and CSY conceptualised and designed and study and wrote the manuscript. SB designed the statistical analysis plan and power estimation. GD contributed to the brain imaging methods and analysis and framing the study objectives. All authors read and approved the final manuscript.

For peer review only

References:

1. Hales CN, Barker DJ. The thrifty phenotype hypothesis. Br Med Bull. 2001;60–5.

2. Fleming TP, Watkins AJ, Velazquez MA, Mathers JC, Prentice AM, Stephenson J, et al. Origins of lifetime health around the time of conception: causes and consequences. Lancet Lond Engl. 2018 May 5;391(10132):1842–52.

3. Schlotz W, Phillips DI. Fetal origins of mental health: evidence and mechanisms. Brain Behav Immun. 2009;23(7):905–16.

4. Pagliaccio D, Alqueza KL, Marsh R, Auerbach RP. Brain Volume Abnormalities in Youth at High Risk for Depression: Adolescent Brain and Cognitive Development Study. J Am Acad Child Adolesc Psychiatry. 2019 Oct 18;

5. Zhang W, Sweeney JA, Yao L, Li S, Zeng J, Xu M, et al. Brain structural correlates of familial risk for mental illness: a meta-analysis of voxel-based morphometry studies in relatives of patients with psychotic or mood disorders. Neuropsychopharmacol Off Publ Am Coll Neuropsychopharmacol. 2020 Jul;45(8):1369–79.

6. Kampman O, Poutanen O. Can onset and recovery in depression be predicted by temperament? A systematic review and meta-analysis. J Affect Disord. 2011 Dec;135(1–3):20–7.

7. Fernandes M, Srinivasan K, Menezes G, Ramchandani PG. Prenatal depression, fetal neurobehavior, and infant temperament: Novel insights on early neurodevelopment from a socioeconomically disadvantaged Indian cohort. Dev Psychopathol. 2018 Aug;30(3):725–42.

8. Veena SR, Gale CR, Krishnaveni GV, Kehoe SH, Srinivasan K, Fall CH. Association between maternal nutritional status in pregnancy and offspring cognitive function during childhood and adolescence; a systematic review. BMC Pregnancy Childbirth. 2016 Aug 12;16:220.

9. Yajnik CS, Deshmukh US. Maternal nutrition, intrauterine programming and consequential risks in the offspring. Rev Endocr Metab Disord. 2008 Sep;9(3):203–11.

10. Schulz LC. The Dutch Hunger Winter and the developmental origins of health and disease. Proc Natl Acad Sci U S A. 2010 Sep 28;107(39):16757–8.

11. Li C, Lumey LH. Exposure to the Chinese famine of 1959-61 in early life and long-term health conditions: a systematic review and meta-analysis. Int J Epidemiol. 2017 Aug 1;46(4):1157–70.

12. Susser ES, Lin SP. Schizophrenia after prenatal exposure to the Dutch Hunger Winter of 1944-1945. Arch Gen Psychiatry. 1992 Dec;49(12):983–8.

13. Brown AS, Susser ES, Lin SP, Neugebauer R, Gorman JM. Increased risk of affective disorders in males after second trimester prenatal exposure to the Dutch hunger winter of 1944-45. Br J Psychiatry J Ment Sci. 1995 May;166(5):601–6.

14. He P, Chen G, Guo C, Wen X, Song X, Zheng X. Long-term effect of prenatal exposure to malnutrition on risk of schizophrenia in adulthood: Evidence from the Chinese famine of 1959-1961. Eur Psychiatry J Assoc Eur Psychiatr. 2018 Jun;51:42–7.

15. Ben-Shlomo Y, Kuh D. A life course approach to chronic disease epidemiology: conceptual models, empirical challenges and interdisciplinary perspectives. Int J Epidemiol. 2002;31–2.

16. Yajnik CS, Deshmukh US. Fetal programming: maternal nutrition and role of one-carbon metabolism. Rev Endocr Metab Disord. 2012 Jun;13(2):121–7.

17. Sukumar N, Rafnsson SB, Kandala N-B, Bhopal R, Yajnik CS, Saravanan P. Prevalence of vitamin B-12 insufficiency during pregnancy and its effect on offspring birth weight: a systematic review and meta-analysis. Am J Clin Nutr. 2016 May;103(5):1232–51.

18. Rogne T, Tielemans MJ, Chong MF-F, Yajnik CS, Krishnaveni GV, Poston L, et al. Associations of Maternal Vitamin B12 Concentration in Pregnancy With the Risks of Preterm Birth and Low Birth Weight: A Systematic Review and Meta-Analysis of Individual Participant Data. Am J Epidemiol. 2017 Feb 1;185(3):212–23.

19. Godbole K, Deshmukh U, Yajnik C. Nutrigenetic determinants of neural tube defects in India. Indian Pediatr. 2009 Jun;46(6):467–75.

For peer review only

20. Sable P, Dangat K, Kale A, Joshi S. Altered brain neurotrophins at birth: consequence of imbalance in maternal folic acid and vitamin B(1)(2) metabolism. Neuroscience. 2011;190–127.

21. Sable P, Kale A, Joshi A, Joshi S. Maternal micronutrient imbalance alters gene expression of BDNF, NGF, TrkB and CREB in the offspring brain at an adult age. Int J Dev Neurosci. 2014;34–24.

22. Magnus P. The Norwegian Mother and Child Cohort Study (MoBa)–new research possibilities. Vol. 17. Norsk epidemiologi; 2007.

23. Jacka FN, Ystrom E, Brantsaeter AL, Karevold E, Roth C, Haugen M, et al. Maternal and early postnatal nutrition and mental health of offspring by age 5 years: a prospective cohort study. J Am Acad Child Adolesc Psychiatry. 2013;52–10.

24. Roza SJ, van Batenburg-Eddes T, Steegers EA, Jaddoe VW, Mackenbach JP, Hofman A, et al. Maternal folic acid supplement use in early pregnancy and child behavioural problems: the Generation R Study. Br J Nutr. 2010;103–03.

25. Schlotz W, Jones A, Phillips DI, Gale CR, Robinson SM, Godfrey KM. Lower maternal folate status in early pregnancy is associated with childhood hyperactivity and peer problems in offspring. J Child Psychol Psychiatry. 2010;51–5.

26. Ars CL, Nijs IM, Marroun HE, Muetzel R, Schmidt M, Steenweg-de Graaff J, et al. Prenatal folate, homocysteine and vitamin B12 levels and child brain volumes, cognitive development and psychological functioning: the Generation R Study. Br J Nutr. 2016 Jan 22;1–9.

27. Suren P, Roth C, Bresnahan M, Haugen M, Hornig M, Hirtz D, et al. Association between maternal use of folic acid supplements and risk of autism spectrum disorders in children. JAMA. 2013;309–6.

28. Steenweg-de Graaff J, Ghassabian A, Jaddoe VW, Tiemeier H, Roza SJ. Folate concentrations during pregnancy and autistic traits in the offspring. The Generation R Study. Eur J Public Health. 2015;25–3.

29. Bonilla C, Lawlor DA, Taylor AE, Gunnell DJ, Ben-Shlomo Y, Ness AR, et al. Vitamin B-12 status during pregnancy and child's IQ at age 8: a Mendelian randomization study in the Avon longitudinal study of parents and children. PloS One. 2012;7(12):e51084.

30. Samuel TM, Duggan C, Thomas T, Bosch R, Rajendran R, Virtanen SM, et al. Vitamin B(12) intake and status in early pregnancy among urban South Indian women. Ann Nutr Metab. 2013;62(2):113–22.

31. Pathak P, Kapil U, Yajnik CS, Kapoor SK, Dwivedi SN, Singh R. Iron, folate, and vitamin B12 stores among pregnant women in a rural area of Haryana State, India. Food Nutr Bull. 2007 Dec;28(4):435–8.

32. Yajnik CS, Deshpande SS, Jackson AA, Refsum H, Rao S, Fisher DJ, et al. Vitamin B12 and folate concentrations during pregnancy and insulin resistance in the offspring: the Pune Maternal Nutrition Study. Diabetologia. 2008 Jan;51(1):29–38.

33. Dwarkanath P, Barzilay JR, Thomas T, Thomas A, Bhat S, Kurpad AV. High folate and low vitamin B-12 intakes during pregnancy are associated with small-for-gestational

For peer review only

age infants in South Indian women: a prospective observational cohort study. Am J Clin Nutr. 2013 Dec;98(6):1450–8.

34. Bhate V, Deshpande S, Bhat D, Joshi N, Ladkat R, Watve S, et al. Vitamin B12 status of pregnant Indian women and cognitive function in their 9-year-old children. Food Nutr Bull. 2008 Dec;29(4):249–54.

35. Kumaran K, Yajnik P, Lubree H, Joglekar C, Bhat D, Katre P, et al. The Pune Rural Intervention in Young Adolescents (PRIYA) study: design and methods of a randomised controlled trial. BMC Nutr [Internet]. 2017 Dec [cited 2019 Jan 22];3(1). Available from: http://bmcnutr.biomedcentral.com/articles/10.1186/s40795-017-0143-5

36. Wechsler D. Wechsler adult intelligence scale--. 1955;

37. Wechsler D. Wechsler memory scale. 1945;

38. Bean J. Rey auditory verbal learning test, Rey AVLT. Encycl Clin Neuropsychol. 2011;2174–5.

39. Sheehan DV, Lecrubier Y, Sheehan KH, Amorim P, Janavs J, Weiller E, et al. The Mini-International Neuropsychiatric Interview (MINI): the development and validation of a structured diagnostic psychiatric interview for DSM-IV and ICD-10. J Clin Psychiatry. 1998;

40. Lanka P, Deshpande G. Combining Prospective Acquisition CorrEction (PACE) with retrospective correction to reduce motion artifacts in resting state fMRI data. Brain Behav. 2019;9(8):e01341.

41. Lanka P, Deshpande G. Resting state fMRI data from subjects scanned with the EPI-PACE (Echoplanar Imaging–Prospective Acquisition CorrEction) sequence. Data Brief. 2018;20:2072–5.

For peer review only

For peer review only

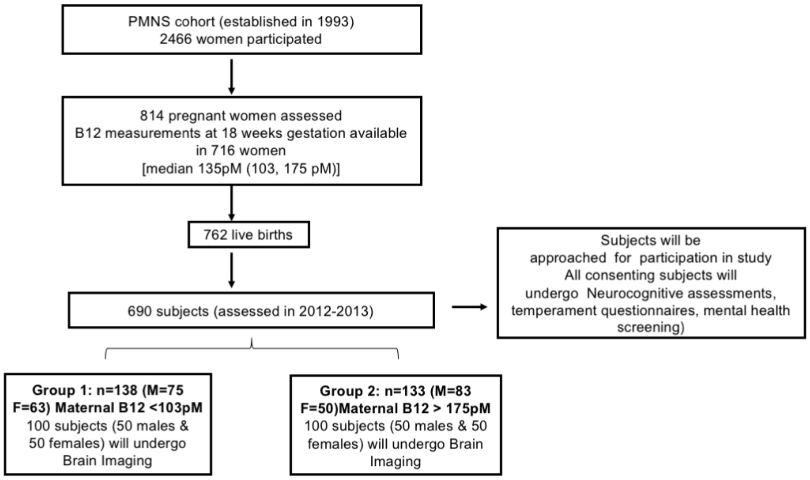

Figure 1 - Selection of subjects from the PMNS cohort for the study

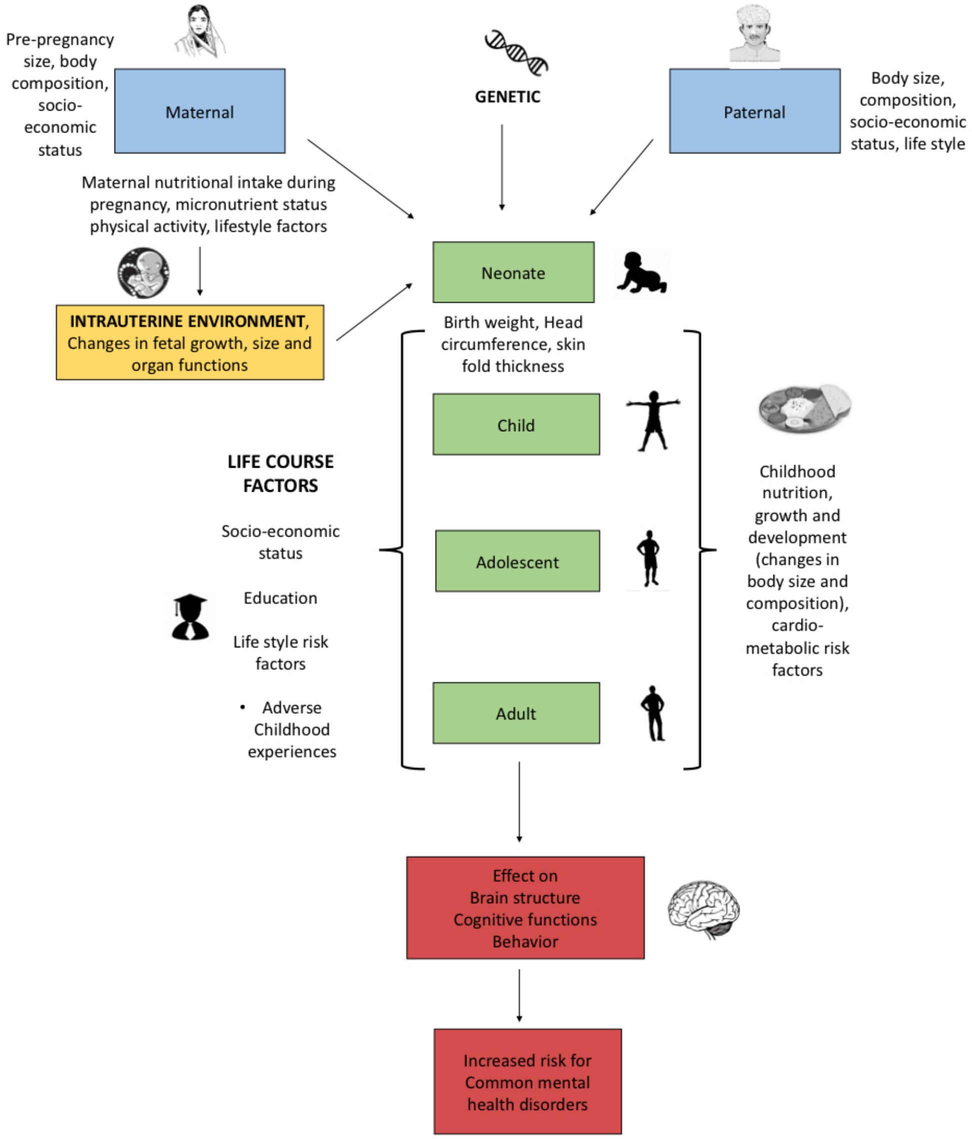

Figure 2 – Understanding neurocognitive development using a life course approach

Supplementary file - Maternal characteristics at 18 and 28 week of pregnancy and birth characteristics of the PMNS cohort

	n	Median (Q1, Q3)
**Maternal nutritional variables at 18 wks pregnancy**		
Total calorie intake (kcal)	796	1736.5 (1402.4,2074.3)
Plasma B12 (pmol/L)	716	135.1 (103.4,175.3)
Red cell folate (ng/mL)	698	388.0 (306,490.25)
Plasma Homocysteine (micromol/L)	717	8.2 (6.8,10.4)
Serum Ferritin (microgm/L)	745	13.0 (8,23)
Serum vitamin C (micromol/l)	764	9.0 (2,30)
**Maternal nutritional variables at 28 wks pregnancy**		
Total calorie intake (kcal)	749	1623.8 (1318.8,1974.8)
Plasma B12 (pmol/L)	617	421.0 (326,556.5)
Red cell folate (ng/mL)	653	8.6 (6.7,10.8)
Plasma Homocysteine (micromol/L)	651	11.0

For peer review only

Supplementary file - Maternal characteristics at 18 and 28 week of pregnancy and birth characteristics of the PMNS cohort

		(7,20)
Serum Ferritin (microgm/L)	687	4.0<br>(1,18)
Serum vitamin C (micromol/l)	749	1623.8<br>(1318.8,1974.8)
**Birth characteristics**		
Birth weight adjusted to 40 wks (gms)	718	2722.2<br>(2509.8,2948.0)
Length at birth adjusted to 40 wks (cms)	708	48.2<br>(47.0,49.3)
Head circ at birth adjusted to 40 wks	709	33.4<br>(32.5,34.2)

For peer review only

Maternal vitamin B12, folate during pregnancy and neurocognitive outcomes in young adults of the Pune Maternal Nutrition Study (PMNS) prospective birth cohort: Study protocol

Journal:	*BMJ Open*
Manuscript ID	bmjopen-2020-046242.R1
Article Type:	Protocol
Date Submitted by the Author:	03-May-2021
Complete List of Authors:	Behere, Rishikesh; KEM Hospital Pune Research Centre, Diabetes Unit<br>Deshpande, Gopikrishna; Auburn University, AU MRI Research Center, Department of Electrical and Computer Engineering, Department of Psychological Sciences, Center for Neuroscience<br>Bandyopadhyay, Souvik; Indian Institute of Public Health<br>Yajnik, Chittaranjan; KEM Hospital Pune, Diabetes Unit, KEMHRC
**Primary Subject Heading**:	Public health
Secondary Subject Heading:	Public health
Keywords:	NUTRITION & DIETETICS, Magnetic resonance imaging < RADIOLOGY & IMAGING, PUBLIC HEALTH

SCHOLARONE™
Manuscripts

I, the Submitting Author has the right to grant and does grant on behalf of all authors of the Work (as defined in the below author licence), an exclusive licence and/or a non-exclusive licence for contributions from authors who are: i) UK Crown employees; ii) where BMJ has agreed a CC-BY licence shall apply, and/or iii) in accordance with the terms applicable for US Federal Government officers or employees acting as part of their official duties; on a worldwide, perpetual, irrevocable, royalty-free basis to BMJ Publishing Group Ltd ("BMJ") its licensees and where the relevant Journal is co-owned by BMJ to the co-owners of the Journal, to publish the Work in this journal and any other BMJ products and to exploit all rights, as set out in our licence.

The Submitting Author accepts and understands that any supply made under these terms is made by BMJ to the Submitting Author unless you are acting as an employee on behalf of your employer or a postgraduate student of an affiliated institution which is paying any applicable article publishing charge ("APC") for Open Access articles. Where the Submitting Author wishes to make the Work available on an Open Access basis (and intends to pay the relevant APC), the terms of reuse of such Open Access shall be governed by a Creative Commons licence – details of these licences and which Creative Commons licence will apply to this Work are set out in our licence referred to above.

Other than as permitted in any relevant BMJ Author's Self Archiving Policies, I confirm this Work has not been accepted for publication elsewhere, is not being considered for publication elsewhere and does not duplicate material already published. I confirm all authors consent to publication of this Work and authorise the granting of this licence.

For peer review only

Maternal vitamin B12, folate during pregnancy and neurocognitive outcomes in young adults of the Pune Maternal Nutrition Study (PMNS) prospective birth cohort: Study protocol

Rishikesh V. Behere[1*], Gopikrishna Desphande[2], Souvik Bandyopadhyay[3], Chittaranjan S Yajnik[4]

1- India Alliance Intermediate Fellow, KEM Hospital Research Center, Pune, India

2 - Professor, AU MRI Research Center, Department of Electrical and Computer Engineering, Department of Psychological Sciences, Center for Neuroscience, Auburn University, Alabama, USA; Alabama Advanced Imaging Consortium, Birmingham, Alabama, USA

3 – Indian Institute of Public Health, Hyderabad, Public Health Foundation of India, Hyderabad, India.

4 - Director, Diabetes Unit, KEM Hospital Research Center, India

[*] **Corresponding Author**

Dr. Rishikesh V. Behere

India Alliance Intermediate Fellow

Diabetes Unit

KEM Hospital Research Center

Pune, India

Email: rvbehere@gmail.com

Ph no: +912026061958

For peer review only

Abstract:

Introduction:

The Developmental origins of Health and Disease (DOHaD) hypothesis, proposes that intrauterine and early life exposures significantly influence fetal development and risk for disease in later life. Evidence from prospective birth cohorts suggests a role for maternal B12 and folate in influencing neurocognitive outcomes in the offspring. In the Indian setting, B12 deficiency is common during pregnancy while rates of folate deficiency are lower. The long term influences of maternal nutrition during pregnancy on adult neurocognitive outcomes have not been examined. The Pune Maternal Nutrition Study (PMNS) is a pre-conceptional birth cohort into its 24th year and is considered a unique resource to study the DOHaD hypothesis. We found an association between maternal B12 status in pregnancy and child's neurocognitive status at 9 years of age. We now plan to assess neurocognitive function and MRI measurements of brain structural-functional connectivity at young adult age to study its association with maternal nutritional exposures during pregnancy.

Methods and analysis:

As part of ongoing prospective follow up in young adults of the PMNS at the Diabetes Unit, KEM Hospital Research Center, Pune India, the following measurements will be done: Neurocognitive performance (Standardized tests of Intelligence, verbal and visual memory, attention and executive functions), temperament (Adult temperament questionnaire), psychopathology (Brief symptom inventory and clinical interview on Mini Neuropsychiatric Interview 7.0). Brain Magnetic Resonance Imaging (MRI) for structural T1, resting-state functional connectivity and diffusion tensor imaging will be performed on a subset of the cohort (selected based on exposure to a lower or higher maternal B12 status at 18 weeks of pregnancy).

For peer review only

Ethics and dissemination:

The study is approved by Institutional ethics committee of KEM Hospital Research Center, Pune. The results will be shared at national and international scientific conferences and published in peer reviewed scientific journals.

Trial Registration: ClinicalTrials.gov ID: NCT03096028

Keywords: maternal nutrition, vitamin B12, neurocognitive development, brain MRI, birth cohort,

Strengths and Limitations:

1. Investigation of structural and functional neurodevelopment in the young adults in the PMNS in relation to intrauterine exposure to vitamin B12 and folate
2. A unique dataset of intergenerational life course exposures (physical growth, nutrition, childhood adversity) to investigate pathways of neurocognitive development.
3. Comprehensive behavioural measures and neuroimaging techniques (structural and functional)
4. Cross-sectional MRI measurements instead of trajectories of brain growth.
5. First life-course study in LMIC to evaluate associations of early life exposures with brain outcomes in adult age

For peer review only

Introduction:

The Developmental origins of Health and Disease (DOHaD) hypothesis(1,2) proposes that early life exposures along with post-natal environmental factors (childhood experiences, education, nutrition, infections, lifestyle, socio-economic conditions) determine risk for disease in later life. Recent investigations suggest an early life origin of neurodevelopmental disorders (autism spectrum disorders, depression, schizophrenia)(3). Early intrauterine insults can impact development of brain areas that show high neuronal growth such as hippocampus and cortical areas (4). Later life factors influence synaptic pruning and myelination (5). Deranged Brain structural and functional connectivity within and between hippocampal, frontal and subcortical networks is known to be associated with impaired neurocognitive functioning and neurodevelopmental disorders (6,7).

The growing fetus is wholly dependent on the mother for its nutrition. Deficiency of various micronutrients in the mother during critical phases of foetal development can affect organ structure and function, hence programming the risk for chronic disease in later life (fetal programming) (8). Recently there is considerable interest in the role of maternal vitamin B12 and folate in fetal programming.

Vitamin B12 is important for nerve myelination and its deficiency is associated with subacute combined degeneration of spinal cord, depression and dementia(9). The role of folate deficiency in risk for neural tube defects is well known. Both these vitamins influence the one carbon metabolism cycle(10). In the presence of vitamin B12 deficiency, folate is trapped in its unusable methyl form and methyl groups are unavailable for further biochemical reactions. This interferes with important cell processes such as purine pyrimidine synthesis, DNA repair, and epigenetic regulation (DNA methylation). Unavailability of methyl groups prevents conversion of homocysteine to S-Adenosyl Methionine leading to hyperhomocysteinemia which can adversely affect vascular endothelial and placental function. Additionally an imbalance of these two vitamins (low vitamin B12-high folate) can

For peer review only - http://bmjopen.bmj.com/site/about/guidelines.xhtml

impact cell processes. Folate supplements are available as folic acid which may competitively inhibit natural folates and needs to be metabolised in the liver. Indiscriminate supplementation in the presence of B12 deficiency leads to accumulation of unmetabolized folic acid (UMFA) which are known to have neurotoxic effects(11).

A recent systematic review found some evidence linking low micronutrient status (vitamin B12, and folate) with poorer offspring cognitive functions(12). Observations from large prospective cohort studies such as the Norwegian mother and child birth cohort (MoBa)(13) and Generation R support association of poor maternal folate status with poorer neurocognitive functioning, greater childhood behavioral problems(14–16), smaller brain volumes(17) and risk for autism(18,19). The Avon Longitudinal Study on Parents And Children (ALSPAC) reported association between low maternal dietary intake of vitamin B12 and lower IQ scores at 8 year and genetic determinants of B12 (FUT2gene) (20).

In India, vitamin B12 deficiency is widely prevalent in women in the reproductive age group while rates of folate deficiency are low (21–23). The vitamin B12 deficiency is attributable to practice of vegetarianism and low socio-economic status. Public health policy mandates only iron-folic acid supplementation in adolescents and pregnant women. It is also a common clinical practice to supplement high dose folic acid (5mg) to pregnant women to prevent NTD. This scenario inadvertently promotes an imbalance between vitamin B12 and folate status during pregnancy(24,25) which could enhance risk of adverse health outcomes in the offspring including aberrant neurodevelopment. Animal experiments performed in India, have examined the role of a maternal B12-folate micronutrient imbalance and show that offspring dams of mothers fed on a lowB12-highfolate diet have poorer spatial memory functions, lower brain hippocampal weight, reduced neurotrophic factor BDNF and reduced TRK gene expression(26,27). Prospective cohort studies from India support the associations of lower maternal vitamin B12 or folate with higher risk for NTD and poorer cognitive functions in offspring in early (30-48 months) and late (9-10 years) childhood. However, no study in India has examined long term effects of maternal vitamin and folate during pregnancy on offspring neurodevelopmental outcomes in young adulthood.

For peer review only

The Pune Maternal Nutrition Study (PMNS) is a pre-conceptional birth cohort with serial data on maternal nutritional status, birth outcomes and offspring nutrition, physical growth and development. The low B12-high folate dietary pattern was associated with adverse metabolic outcomes in the offspring at 6 years(23). We have earlier demonstrated in this cohort that exposure to a low maternal B12 was associated with poor cognitive functioning in the offspring at 9 years of age(28). The offspring of this cohort are now in their early 20's and this provides a unique opportunity to examine the long-term effects of maternal nutrition status during pregnancy on neurocognitive outcomes in young adulthood.

Aims and objectives:

The key aim of the study is to examine whether maternal micronutrient status during pregnancy (B12, folate) is associated with neurocognitive performance, structural and functional connectivity and risk for common mental disorders in young adult offspring of the PMNS birth cohort.

Primary objectives:

1. To examine associations between maternal vitamin B12, folate, and their interaction at 18 and 28 weeks of pregnancy with neurocognitive outcomes (neuropsychological test performance) and risk for common mental disorders (temperament, presence of depression or anxiety disorders) in young adulthood; controlling for the effects of other nutrients (calorie, protein and fat intake, and levels of vitamin C,D, ferritin) and later life developmental influences (education, adverse childhood experiences, offspring nutrition, physical growth and development, metabolic status).
2. To examine the association of maternal nutritional status during pregnancy with brain structure (grey matter volume and cortical thickness), and structural/functional

For peer review only - http://bmjopen.bmj.com/site/about/guidelines.xhtml

connectivity (using Diffusion Tensor Imaging – DTI and resting state functional Magnetic Resonance Imaging – rsfMRI, respectively).

3. To test the causality of association between maternal vitamin B12, folate, and neurocognitive outcomes using maternal and offspring genetic determinants of vitamin B12.

Hypotheses:

We hypothesise that a lower maternal B12, higher folate, higher homocysteine nutrient pattern during pregnancy adversely impacts development of brain regions, as well as structural/functional networks (hippocampal and frontal-subcortical connections) involved in cognitive processing (memory, executive functions) and this reflects in poorer neurodevelopmental outcomes (lower neuropsychological test performance, adverse temperamental traits and higher risk for common mental disorders) in the young adult offspring.

Methods and Analysis:

Study setting:

Pune Maternal Nutrition Study:

The PMNS is a pre-conceptional cohort, established in 6 villages of Pune district, Maharashtra state, India in 1993 in collaboration with Dr DJP Barker and Dr Caroline Fall and is considered a unique resource in the world to examine the DOHaD paradigm. Initially, 2466 women (F0 generation) in the reproductive age group participated in the study. 814 women who became pregnant in the period June 1994 to April 1996 were enrolled for the study. Of these 797 pregnancies (with singleton pregnancy and period of gestation less than 21 weeks) were included. 762 live births occurred (F1 generation). This cohort has data on pre-

For peer review only - http://bmjopen.bmj.com/site/about/guidelines.xhtml

conceptional parental size, maternal nutrition and size at birth of the offspring. Maternal micronutrients vitamin B12, red cell folate and ferritin have been measured from blood samples collected at 18 and 28 weeks of gestation. Vitamin B12 levels were measured using the microbiological assay technique and red cell folate and ferritin by radioimmunoassay(23,29). Children are being regularly followed up for growth and development (body size and DXA body composition), nutrition, and cardio-metabolic risk factors (6,12&18 years of age). Biological samples have been collected and stored in a bio-repository and are available for assessments. The other strengths of this cohort are good community participation and follow up rates of over 90% over the years. At 18 years follow up, 690 of the original 762 live births in the cohort were followed up.

The participants (F1 generation) of the cohort are also part of a randomized controlled trial (Pune Rural Intervention in Young Adolescents, PRIYA) (30) to examine intergenerational effects of micronutrient supplementation on various health outcomes. In 2012 at the age of 17 years 557 eligible members were randomized as part of the nutrition intervention trial into one of 3 groups 1) 2 micrograms/day of vitamin B12, 2) 2 micrograms/day of Vitamin B12 with UNIMAPP multivitamins and 20g milk powder 3) placebo**.** The trial was completed in the boys in December 2017 and in the girls in September 2019. The effects of later life intervention will be adjusted during statistical analysis. (Figure 1 – Depicts time line of measurements in the PMNS cohort).

Subject selection:

All subjects of the PMNS cohort (n=690) will be invited for participation in the study (Figure 2) subject to following inclusion criteria: 1) Maternal B12, folate levels measured during pregnancy 2) Serial measurements done at 6,12 and 18 years of age. Neurocognitive assessments, temperament and mental health assessments will be performed in all consenting subjects. Brain MRI will be performed on a subset of the cohort selected based on exposure to maternal B12 levels at 18 weeks of gestation. Median B12 level at 18-week

For peer review only

gestation was 153pM (103,175 pM being the 1st and 4th quartiles). Description of maternal characteristics at 18 and 28-week gestation and offspring birth characteristics are provided in the supplementary material - 1. Of the 690 participants still in follow up, 152 subjects (78 males, 74 females) whose mothers had plasma vitamin B12 concentration <103pM and 154 subjects (88 males, 66 females) whose mothers had plasma vitamin B12 concentration >175pM will be eligible for participation in the brain MRI scans. 100 participants each from these two groups (total of 200 subjects) will be randomly selected..

For peer review only

Assessments:

The exposures and outcome measurements are summarised in Table – 1.

Behavioural assessments:

These include 1) Standardized neurocognitive test batteries - Wechsler's adult intelligence scale (WAIS-IV), Wechsler Memory Scale (WMS), Auditory verbal learning test (AVLT), Color trail test. 2) Adult Temperament Questionnaire (ATQ) 3) Mental health assessments - Brief symptom inventory (BSI), Mini Neuropsychiatric Interview (MINI plus 7.0). Further details of behavioural assessments are provided in the supplementary material-2. 4) Early life stress. - WHO Adverse Childhood Experiences International Questionnaire (ACE-IQ).

	**Exposures**	**Tests**	**Variables measured**
**1.**	Maternal Micronutrient status and homocysteine at 18 and 28 wks pregnancy	Biochemical assays	Vitamin B12, red cell folate, homocysteine
	**Outcomes**	**Tests**	**Variables measured**
1.	Neurocognitive performance	WAIS-IV<br>WMS<br>AVLT<br>Color Trails test	Verbal comprehension, perceptual reasoning, working memory, processing speed, Full scale IQ.<br>Immediate Memory, delayed memory and working memory.<br>Errors in Immediate and delayed recall

For peer review only - http://bmjopen.bmj.com/site/about/guidelines.xhtml

			Time taken and errors for Color trails test A & B.
2.	Temperament	Adult Temperament Questionnaire	Negative affect, extraversion, orienting sensitivity, effortful control
3.	Common mental disorders	Brief Symptom Inventory<br>Mini Neuropsychiatric Interview 7.0 for DSM 5 diagnosis	Global severity index<br>DSM5 diagnosis of past or current Major Depressive disorder, suicidality, Panic disorder, Agoraphobia, Social anxiety disorder, generalised anxiety disorder
5.	Brain Imaging	Structural T1 MPRAGE<br>Resting state fMRI<br>Diffusion tensor imaging	Cortical volume and thickness<br>Resting state functional connectivity<br>Tractography, FA and MD values in relevant white matter tracts
	**Life-course variables**		
	Birth		Birth weight, length, head circumference, sum of skin folds
	Later life growth and development		Height, BMI, head circumference sum of skin folds at 6, 12, 18 years
	Later life nutrition		Hemoglobin, vitamin B12, folate, homocysteine, ferritin, glucose, lipids at 6,12,18 years
	Socio-environmental		Mothers education, fathers education, standard of living index, adverse childhood experiences

Table -1 Exposures and Outcomes defined in the study

Magnetic resonance imaging:

Anatomical data using structural 3D T1 weighted MPRAGE (magnetization prepared rapid gradient echo) sequence, resting state fMRI (rs-fmri) with 2D gradient-echo EPI (echo planar imaging) sequence, and Diffusion Tensor Imaging (DTI) using RESOLVE sequence will be acquired on a 3 Tesla Siemens Skyra scanner.

For peer review only

Image acquisition

For structural imaging, 3D T1-weighted MPRAGE images will be acquired (TR=2300 ms, TE=2.26 ms, TI=900 ms, Flip angle=8 degrees, slice thickness=1 mm, in-plane resolution= 1×1 mm^2, GRAPPA parallel imaging with acceleration factor of 2, 192 slices, FOV readout=256 mm, FOV phase encoding= 100%, and acquisition time of 5 min and 21 s).

For functional imaging, T2*-weighted echo-planar imaging sequence will be used to acquire BOLD fMRI data while participants are resting with eyes open and fixated, and not thinking about anything specific. Thirty six axial slices will be acquired covering the whole brain with a slice thickness of 3 mm, in-plane resolution of 3×3 mm^2, TR=2000 ms; TE=30 ms; flip angle=90^0, FOV readout=256 mm, FOV phase encoding= 100%, GRAPPA parallel imaging with acceleration factor of 2, and 300 measurements with an acquisition time of 10 min and 8 s. PACE (prospective acquisition correction) motion correction will be employed in this sequence. (31,32)

Diffusion-weighted images will be acquired using the RESOLVE (readout segmentation of long variable echo-trains) sequence given its robustness against susceptibility artifacts. Scan parameters for RESOLVE will be: Thirty-six axial slices covering the whole brain with a slice thickness of 3 mm, in-plane resolution of 1.4×1.4 mm^2, TR=5460 ms; TE=73/109 ms; flip angle=180^0, FOV readout=220 mm, FOV phase encoding= 100%, GRAPPA parallel imaging with acceleration factor of 2, 30 diffusion directions, two diffusion weightings (b-values) of 0 and 1000 s/mm^2 and an acquisition time of 14 min and 19 s.

All MRI images will be visually inspected for quality assessment and reviewed by a radiologist to rule out any structural abnormalities.

Data management:

For peer review only - http://bmjopen.bmj.com/site/about/guidelines.xhtml

Data will be stored at the Diabetes Unit, KEM Hospital Research Center, Pune, India. Data collected will be first entered in a paper clinical record form and double entered into an open clinical platform on a computer. Data is downloaded and checked for extreme or missing values. Data on maternal micronutrient status during pregnancy (nutrition intake, circulating micronutrients) and intermediate variables (birth size, childhood nutrition, growth and development, metabolic factors (insulin resistance, glycemic status, adiposity), socio demographic variables and socio-economic status have been assessed previously on the cohort. These will be extracted and used for analysis.

Statistical methods:

Power estimation:

There are 690 members of the cohort available for assessments. Assuming a 80% participation rate (from experience of previous studies on the cohort and accounting for migration) we estimate that 550 subjects would be recruited for the study. Using a test at 5% significance level, the study will have 80% power to detect an association of 0.11 standard deviation of continuous outcome (eg. neurocognitive test score) per standard deviation of continuous exposure (eg. maternal vitaminB12 level). Study of 200 subjects (100 in each group) for Brain imaging will have 80% power for an effect size 0.3 and significance at $p < 0.05$ for detecting difference between the two maternal B12 exposure groups.

Plan of Data analysis:

For peer review only - http://bmjopen.bmj.com/site/about/guidelines.xhtml

Objective 1: This examines the association between intrauterine nutritional exposures and neurocognitive outcomes in young adult age:

Our primary exposure of interest is maternal vitamin B12 and red cell folate levels. Due to lack of reliable clinical cutoffs for these micronutrients during pregnancy for our population, we prefer to use the distribution within population to classify participants. Such an approach has been used earlier to report metabolic outcomes in the offspring in this cohort (23). Distribution of outcome variables such as neurocognitive performance scores will be visualized by using different plotting techniques such as box plot and scatter plot. Univariate analysis (Pearson' correlation and linear regression) will be performed to examine the relationships between the primary exposures, life course variables and neurocognitive outcomes (table -1). We expect to construct multivariate linear (if outcome is continuous) or logistic regression (categorical outcome) models to examine associations of our primary exposures with neurocognitive outcomes and extend the models to examine potential modifying and mediating effects of life-course variables on the associations. Finally, we will adjust for the B12 intervention status (B12 intervention received yes or no) in the PRIYA trial. We will follow this broad scheme of analysis. Depending on observed univariate associations we may construct additional multivariate models.

Given the multitude of factors and possible collinearity we will use dimensionality reduction techniques (such as Principal Component Analysis - PCA). The components might be used to construct path analytical models for exploring associations between outcomes.

Objective 2: This examines the association between maternal nutritional exposures and brain structural functional connectivity:

1. Structural Brain MRI analysis
    a. Voxel-based Morphometry on T1 MPRAGE anatomical images will be performed using statistical parametric mapping in Matlab. Pre-processing

For peer review only - http://bmjopen.bmj.com/site/about/guidelines.xhtml

steps will involve co-registration, segmentation. Spatial normalisation will transform all subject data to the same stereotactic space. Gray matter images will be smoothed by convolving with an isotropic Gaussian kernel. Statistical analysis using General Linear Model will be used to identify regions of grey matter concentration that are significantly associated with our exposures of interest.

b. Cortical volume, thickness and hippocampal segmentation: A surface based morphometric technique will be employed to measure cortical volume and thickness. We will use freesurfer software for image analysis. Standard steps include intensity normalisation, skull stripping and removal of non-brain tissues followed by segmentation, topology correction, registration and automated parcellation. Cortical volume and thickness will be computed for 68 regions (34 in each hemisphere) using the Desikan-Kelliany Atlas. The hippocampal subfield segmentation tool in freesurfer will be employed to compute volumes of the various hippocampal structures as per the inbuilt atlas which defines 11 hippocampal subfield structures.

2. Structural Connectivity – Diffusion tractography analysis: Diffusion weighted images will be analysed using the "The Diffusion Toolbox" software tool in the FMRIB Software Library (FSL; http://www.fmrib.ox.ac.uk/fsl/fdt/index.html). After pre-processing including motion correction and eddy current correction, the DTI indices fractional anisotropy (FA), mean diffusivity (MD) and radial diffusivity (RD) will be computed. Structural connectome generation will be performed on the MRItrix3 software. Pre-processing steps will involve denoising and correction for eddy currents. The b0 volume of pre-processed images will be extracted and registered to the T1 image using FSL's BET tool. The fiber orientation dispersion functions (FODs) will be generated for white matter and normalised. Probabilistic streamlines tractography will be performed

For peer review only

by dynamically seeding the normalized white matter FOD's. The whole brain tractogram thus generated will be used for structural connectome generation.

3. Functional Connectivity: CONN software (https://web.conn-toolbox.org/) will be used for the analysis of rs-fMRI data. This will involve standard pre-processing steps such as realignment, normalization to MNI space, elimination of temporal linear trends and regressing out nuisance covariates such as residual motion and physiological artifacts. *aCompCor* will be used for minimizing physiological artifacts. Motion censoring and interpolation will also be used to remove the effects of large frame wise displacements due to head motion. Following pre-processing, mean time series will be extracted from 91 cortical and 15 sub-cortical regions in the FSL Harvard-Oxford atlas, as well as from 26 cerebellar regions in the AAL atlas. These time series will be used in a whole brain functional connectivity analysis employing Pearson's correlation coefficient between the mean time series.

For each modality, the following statistical comparisons will be performed. We will first perform correlations between maternal B12 and folate exposures with brain MRI outcomes to examine for any linear relationships. We will use an analysis of covariance (ANCOVA) model to examine differences in MRI outcomes between low and high maternal B12 groups. Similarly, we will examine for differences between low and high maternal red cell folate groups (selected based on median value of maternal red cell folate) and for any interaction between maternal B12 and folate status. Variance due to subjects' age, gender, total intracranial volume, adverse childhood experiences, will be regressed out of the analyses. Effects of additional factors (such as current B12 status of offspring, education status, socio-economic status) will be examined.

For peer review only

For making inferences from multimodal imaging data, we will adopt a hierarchical approach. Accordingly, we will perform voxel-wise tests for difference in grey matter volume (or cortical thickness). For brain regions which are significant, DTI-based structural connectivity between those regions will be investigated. For regions whose structural connectivity is also significantly different for the contrasts of interest, fMRI-based functional connectivity between such regions will be tested. This strategy assures that the effects of interest have both structural and functional etiologies, and hence are likely to be robust and reproducible.

Objective 3:

This examines causality of association between maternal nutritional factors and neurocognitive outcomes. Mendelian randomization analysis (triangulation and instrumental variable analysis) using genetic determinant of vitaminB12 (TCN2rs1801198, FUT2rs492602) and homocysteine (MTHFRrs1801133) will be performed.

Patient and public involvement:

The present study is a follow up of participants of the Pune Maternal Nutrition Study birth cohort. This cohort was set up in 1993 from six villages within Pune district in India. The cohort has been serially evaluated every 6 years (at 6,12,18 years) and is currently in the 24th year of its follow up. The cohort participants were also part of randomized controlled trial from 2012-2019. We are also following up the children born in the next generation of the cohort. The cohort has a high follow up rate of 90% over the last 18 years. This was possible due to active community engagement and involvement of social workers who are from the same community and interact with cohort participants at regularly. Information on results of our studies are shared with cohort participants through public engagement initiatives.

Ethics and dissemination:

For peer review only - http://bmjopen.bmj.com/site/about/guidelines.xhtml

The study will be carried out as per the ethical guidelines provided by the Indian Council of Medical Research. The study has been approved by the Institutional Ethics Committee of the KEM Hospital Research Center, Pune (KEMHRC/RVM/EC/1249). Study is registered with clinicaltrials.gov (ID-NCT 03096028). The cohort participants will be approached by the field workers who have been following them in the community for last many years. Participants will be provided the study information sheet and an opportunity to discuss their doubts and concerns regarding the study with the investigator and then recruited after they sign a written informed consent.

The data generated from this study will be shared with the scientific community at local, national and international conferences, and published in open access peer reviewed journals. The major findings will be shared with the general public, study participants and their families in lay terms through public engagement initiatives.

The study will help build a database on neurocognitive and mental health outcomes and brain imaging in young adulthood in a pre-conceptional birth cohort. The results of the association of maternal B12 during pregnancy and long term neurodevelopmental outcomes will inform public health actions for nutritional health of adolescent girls and women of reproductive age.

Figure 1 - Understanding neurocognitive development using a life course approach in the PMNS

Figure 2 - Selection of subjects from the PMNS cohort for the study

Competing interests:

The authors declare that they have no competing interests.

For peer review only

Funding:

This work is supported by the DBT/Wellcome Trust India Alliance Fellowship [IA/CPHI/161502665] awarded to Dr Rishikesh V. Behere.

Author's contribution:

RVB and CSY conceptualised and designed the study and wrote the manuscript. SB designed the statistical analysis plan and power estimation. GD contributed to the brain imaging methods and analysis and framing the study objectives. All authors read and approved the final manuscript.

References:

1. Hales CN, Barker DJ. The thrifty phenotype hypothesis. Br Med Bull. 2001;60–5.
2. Fleming TP, Watkins AJ, Velazquez MA, Mathers JC, Prentice AM, Stephenson J, et al. Origins of lifetime health around the time of conception: causes and consequences. Lancet Lond Engl. 2018 May 5;391(10132):1842–52.
3. Schlotz W, Phillips DI. Fetal origins of mental health: evidence and mechanisms. Brain Behav Immun. 2009;23(7):905–16.
4. Gilchrist C, Cumberland A, Walker D, Tolcos M. Intrauterine growth restriction and development of the hippocampus: implications for learning and memory in children and adolescents. Lancet Child Adolesc Health. 2018 Oct;2(10):755–64.
5. Langen CD, Muetzel R, Blanken L, van der Lugt A, Tiemeier H, Verhulst F, et al. Differential patterns of age-related cortical and subcortical functional connectivity in 6-to-10 year old children: A connectome-wide association study. Brain Behav. 2018 Aug;8(8):e01031.
6. Liu J, Chen Y, Stephens R, Cornea E, Goldman B, Gilmore JH, et al. Hippocampal functional connectivity development during the first two years indexes 4-year working memory performance. Cortex. 2021 May;138:165–77.
7. Yoon YB, Shin W-G, Lee TY, Hur J-W, Cho KIK, Sohn WS, et al. Brain Structural Networks Associated with Intelligence and Visuomotor Ability. Sci Rep. 2017 Dec;7(1):2177.
8. Yajnik CS, Deshmukh US. Maternal nutrition, intrauterine programming and consequential risks in the offspring. Rev Endocr Metab Disord. 2008 Sep;9(3):203–11.

For peer review only

9. Misra UK, Kalita J, Das A. Vitamin B12 deficiency neurological syndromes: a clinical, MRI and electrodiagnostic study. Electromyogr Clin Neurophysiol. 2003 Feb;43(1):57–64.

10. Yajnik CS, Deshmukh US. Fetal programming: maternal nutrition and role of one-carbon metabolism. Rev Endocr Metab Disord. 2012 Jun;13(2):121–7.

11. Bailey R, Jun S, Murphy L, Green R, Miller J. High Folate and Vitamin Low B12 Status: Potential Interactions with Cognitive Function Among U.S. Older Adults, NHANES 2011–2014. Curr Dev Nutr. 2020 Jun 1;4(Supplement_2):1189–1189.

12. Veena SR, Gale CR, Krishnaveni GV, Kehoe SH, Srinivasan K, Fall CH. Association between maternal nutritional status in pregnancy and offspring cognitive function during childhood and adolescence; a systematic review. BMC Pregnancy Childbirth. 2016 Aug 12;16:220.

13. Magnus P. The Norwegian Mother and Child Cohort Study (MoBa)–new research possibilities. Vol. 17. Norsk epidemiologi; 2007.

14. Jacka FN, Ystrom E, Brantsaeter AL, Karevold E, Roth C, Haugen M, et al. Maternal and early postnatal nutrition and mental health of offspring by age 5 years: a prospective cohort study. J Am Acad Child Adolesc Psychiatry. 2013;52–10.

15. Roza SJ, van Batenburg-Eddes T, Steegers EA, Jaddoe VW, Mackenbach JP, Hofman A, et al. Maternal folic acid supplement use in early pregnancy and child behavioural problems: the Generation R Study. Br J Nutr. 2010;103–03.

16. Schlotz W, Jones A, Phillips DI, Gale CR, Robinson SM, Godfrey KM. Lower maternal folate status in early pregnancy is associated with childhood hyperactivity and peer problems in offspring. J Child Psychol Psychiatry. 2010;51–5.

17. Ars CL, Nijs IM, Marroun HE, Muetzel R, Schmidt M, Steenweg-de Graaff J, et al. Prenatal folate, homocysteine and vitamin B12 levels and child brain volumes, cognitive development and psychological functioning: the Generation R Study. Br J Nutr. 2016 Jan 22;1–9.

18. Suren P, Roth C, Bresnahan M, Haugen M, Hornig M, Hirtz D, et al. Association between maternal use of folic acid supplements and risk of autism spectrum disorders in children. JAMA. 2013;309–6.

19. Steenweg-de Graaff J, Ghassabian A, Jaddoe VW, Tiemeier H, Roza SJ. Folate concentrations during pregnancy and autistic traits in the offspring. The Generation R Study. Eur J Public Health. 2015;25–3.

20. Bonilla C, Lawlor DA, Taylor AE, Gunnell DJ, Ben-Shlomo Y, Ness AR, et al. Vitamin B-12 status during pregnancy and child's IQ at age 8: a Mendelian randomization study in the Avon longitudinal study of parents and children. PloS One. 2012;7(12):e51084.

21. Samuel TM, Duggan C, Thomas T, Bosch R, Rajendran R, Virtanen SM, et al. Vitamin B(12) intake and status in early pregnancy among urban South Indian women. Ann Nutr Metab. 2013;62(2):113–22.

For peer review only

22. Pathak P, Kapil U, Yajnik CS, Kapoor SK, Dwivedi SN, Singh R. Iron, folate, and vitamin B12 stores among pregnant women in a rural area of Haryana State, India. Food Nutr Bull. 2007 Dec;28(4):435–8.

23. Yajnik CS, Deshpande SS, Jackson AA, Refsum H, Rao S, Fisher DJ, et al. Vitamin B12 and folate concentrations during pregnancy and insulin resistance in the offspring: the Pune Maternal Nutrition Study. Diabetologia. 2008 Jan;51(1):29–38.

24. Behere RV, Yajnik CS. Low vitamin B-12–high folate status in adolescents and pregnant women may have deleterious effects on health of the offspring. Am J Clin Nutr. 2021 Apr 6;113(4):1057–9.

25. Behere RV, Deshmukh AS, Otiv S, Gupte MD, Yajnik CS. Maternal Vitamin B12 Status During Pregnancy and Its Association With Outcomes of Pregnancy and Health of the Offspring: A Systematic Review and Implications for Policy in India. Front Endocrinol. 2021 Apr 12;12:619176.

26. Sable P, Dangat K, Kale A, Joshi S. Altered brain neurotrophins at birth: consequence of imbalance in maternal folic acid and vitamin B(1)(2) metabolism. Neuroscience. 2011;190–127.

27. Sable P, Kale A, Joshi A, Joshi S. Maternal micronutrient imbalance alters gene expression of BDNF, NGF, TrkB and CREB in the offspring brain at an adult age. Int J Dev Neurosci. 2014;34–24.

28. Bhate V, Deshpande S, Bhat D, Joshi N, Ladkat R, Watve S, et al. Vitamin B12 status of pregnant Indian women and cognitive function in their 9-year-old children. Food Nutr Bull. 2008 Dec;29(4):249–54.

29. Rao S, Yajnik CS, Kanade A, Fall CH, Margetts BM, Jackson AA, et al. Intake of micronutrient-rich foods in rural Indian mothers is associated with the size of their babies at birth: Pune Maternal Nutrition Study. J Nutr. 2001 Apr;131(4):1217–24.

30. Kumaran K, Yajnik P, Lubree H, Joglekar C, Bhat D, Katre P, et al. The Pune Rural Intervention in Young Adolescents (PRIYA) study: design and methods of a randomised controlled trial. BMC Nutr [Internet]. 2017 Dec [cited 2019 Jan 22];3(1). Available from: http://bmcnutr.biomedcentral.com/articles/10.1186/s40795-017-0143-5

31. Lanka P, Deshpande G. Combining Prospective Acquisition CorrEction (PACE) with retrospective correction to reduce motion artifacts in resting state fMRI data. Brain Behav. 2019;9(8):e01341.

32. Lanka P, Deshpande G. Resting state fMRI data from subjects scanned with the EPI-PACE (Echoplanar Imaging–Prospective Acquisition CorrEction) sequence. Data Brief. 2018;20:2072–5.

For peer review only

For peer review only

2

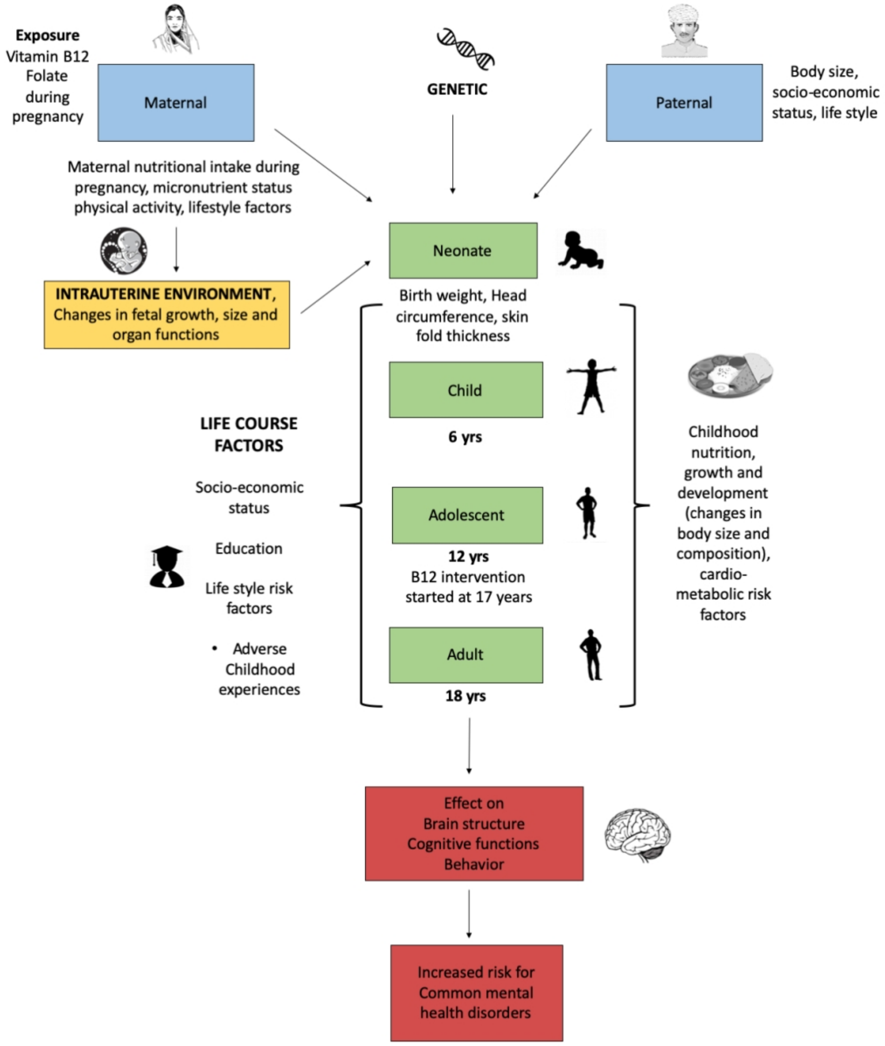

Figure 1 - Understanding neurocognitive development using a life course approach in the PMNS

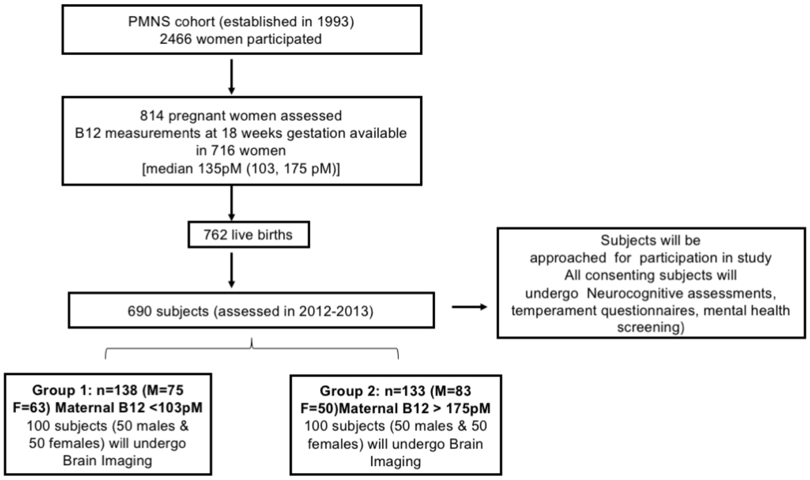

Figure 2 - Selection of subjects from the PMNS cohort for the study

	n	Median (Q1, Q3)
**Maternal nutritional variables at 18 wks pregnancy**		
Total calorie intake (kcal)	796	1736.5 (1402.4,2074.3)
Plasma B12 (pmol/L)	716	135.1 (103.4,175.3)
Red cell folate (ng/mL)	698	388.0 (306,490.3)
Plasma Homocysteine (micromol/L)	717	8.2 (6.8,10.4)
Serum Ferritin (microgm/L)	745	13.0 (8,23)
**Maternal nutritional variables at 28 wks pregnancy**		
Total calorie intake (kcal)	749	1623.8 (1318.8,1974.8)
Plasma B12 (pmol/L)	656	121.5 (93.5 , 156.9)
Red cell folate (ng/mL)	617	421.0 (326,556.5)
Plasma Homocysteine (micromol/L)	653	8.6 (6.7,10.9)
Serum Ferritin (microgm/L)	651	11.0 (7, 20)

For peer review only

Birth characteristics		
Birth weight adjusted to 40 wks (gms)	718	2722.2 (2509.8,2948.0)
Length at birth adjusted to 40 wks (cms)	708	48.2 (47.0,49.3)
Head circ at birth adjusted to 40 wks	709	33.4 (32.5,34.2)

Supplementary material – 1 Maternal characteristics at 18 and 28 week of pregnancy and birth characteristics of the PMNS cohort

For peer review only

Supplementary material - 2.

Details of behavioural assessment of neurocognitive outcomes

a. Neurocognitive assessment:

Standardized battery of neuropsychological tests to assess Intelligence and the domains of executive functions, working memory, and verbal memory.

1. Wechsler's adult intelligence scale (WAIS-IV): Wechsler Adult Intelligence scale (36) (WAIS)-4th edition measures cognitive ability in adults between the ages of 16 years 0 months and 84 years 11 months. The test provides information on the subject's performance on 10 subtests, 4 sub scales i.e. Verbal Comprehension Index scale (VCI), Perceptual Reasoning Index scale (PRI), Working Memory Index scale (WMI) and Processing Speed Index scale (PSI), and gives a final measure of Full-scale IQ (FSIQ).
2. Wechsler's Memory Scale: Wechsler Memory Scale (WMS) (37) measures memory functioning in adults between the ages of 16 years and 90 years. WMS is comprised of eleven sub-tests, and the person's performance is reported on six indices. In this study, six sub-tests will be administered to measure performance on visual immediate memory, visual delayed memory and working memory.
3. Auditory verbal learning test: This is a test of verbal learning and memory (38). The individual is read out a list of 15 words and is asked to recall the words. The list (list A) is initially read out 5 times and recall is tested after each reading. After this, a second list is read out (list B) and recall is tested. Following the recall of list B, recall of list A is immediately tested again. After a 20-minute gap, the delayed recall of list A is tested. The individual is scored based on number of correct answers, omissions, commissions, and total errors.

For peer review only

4. Color trail test: This is a test of executive functions. It involves of 2 parts A & B where subjects have to connect numbered circles from 1 to 25 followed by connecting numbered circles in alternating colors (yellow and pink). Time to complete task and number of errors are recorded. Longer time indicates poor performance.

b. Psychological assessments:

Adult Temperament Questionnaire (ATQ): This 60-item self-report questionnaire is part of Rothbart's scales of temperament. It Yields scores on the domains of negative affect, effortful control, extraversion and orienting sensitivity

c. Mental Health assessment

Brief symptom inventory (BSI) – This is a self or interviewer administered questionnaire that evaluates psychological distress and psychiatric disorders in people. It consists of 53 items covering nine symptom dimensions. Participants rate their feelings about a given item on a 5-point rating scale ranging from 0 (not at all) to 4 (extremely). Rankings characterize the intensity of distress during the past seven days. The test yields score on 9 psychopathology domains and three global indices of distress: Global Severity Index, Positive Symptom Distress Index, and Positive Symptom Total.

MINI (Mini Neuropsychiatric interview plus 7.0): All subjects will be assessed on a structured clinical interview (39) which yields syndromal psychiatric diagnosis based on the DSM-5**.** CMD of interest assessed would be depression and anxiety disorders. All subjects meeting criteria on the MINI will be reassessed by comprehensive clinical mental status examination to confirm the diagnosis. Any subject found to have syndromal psychiatric diagnosis will be offered treatment as per standard clinical protocols.

d. Early life stress: WHO Adverse Childhood Experiences International Questionnaire (ACE-IQ) is a subject rated questionnaire to assess ACE upto age 18 years. Responses are coded

For peer review only

into binary scores which are summated to yield a total ACE-IQ score. A higher score indicates greater ACE.

For peer review only

BMJ Open

Maternal vitamin B12, folate during pregnancy and neurocognitive outcomes in young adults of the Pune Maternal Nutrition Study (PMNS) prospective birth cohort: Study protocol

Journal:	*BMJ Open*
Manuscript ID	bmjopen-2020-046242.R2
Article Type:	Protocol
Date Submitted by the Author:	07-Aug-2021
Complete List of Authors:	Behere, Rishikesh; KEM Hospital Pune Research Centre, Diabetes Unit<br>Deshpande, Gopikrishna; Auburn University, AU MRI Research Center, Department of Electrical and Computer Engineering, Department of Psychological Sciences, Center for Neuroscience<br>Bandyopadhyay, Souvik; Indian Institute of Public Health<br>Yajnik, Chittaranjan; KEM Hospital Pune, Diabetes Unit, KEMHRC
**Primary Subject Heading**:	Public health
Secondary Subject Heading:	Public health
Keywords:	NUTRITION & DIETETICS, Magnetic resonance imaging < RADIOLOGY & IMAGING, PUBLIC HEALTH

SCHOLARONE™
Manuscripts

I, the Submitting Author has the right to grant and does grant on behalf of all authors of the Work (as defined in the below author licence), an exclusive licence and/or a non-exclusive licence for contributions from authors who are: i) UK Crown employees; ii) where BMJ has agreed a CC-BY licence shall apply, and/or iii) in accordance with the terms applicable for US Federal Government officers or employees acting as part of their official duties; on a worldwide, perpetual, irrevocable, royalty-free basis to BMJ Publishing Group Ltd ("BMJ") its licensees and where the relevant Journal is co-owned by BMJ to the co-owners of the Journal, to publish the Work in this journal and any other BMJ products and to exploit all rights, as set out in our licence.

The Submitting Author accepts and understands that any supply made under these terms is made by BMJ to the Submitting Author unless you are acting as an employee on behalf of your employer or a postgraduate student of an affiliated institution which is paying any applicable article publishing charge ("APC") for Open Access articles. Where the Submitting Author wishes to make the Work available on an Open Access basis (and intends to pay the relevant APC), the terms of reuse of such Open Access shall be governed by a Creative Commons licence – details of these licences and which Creative Commons licence will apply to this Work are set out in our licence referred to above.

Other than as permitted in any relevant BMJ Author's Self Archiving Policies, I confirm this Work has not been accepted for publication elsewhere, is not being considered for publication elsewhere and does not duplicate material already published. I confirm all authors consent to publication of this Work and authorise the granting of this licence.

For peer review only

Maternal vitamin B12, folate during pregnancy and neurocognitive outcomes in young adults of the Pune Maternal Nutrition Study (PMNS) prospective birth cohort: Study protocol

Rishikesh V. Behere[1*], Gopikrishna Deshpande[2], Souvik Bandyopadhyay[3], Chittaranjan S Yajnik[4]

1- India Alliance Intermediate Fellow, KEM Hospital Research Center, Pune, India

2 - Professor, AU MRI Research Center, Department of Electrical and Computer Engineering, Department of Psychological Sciences, Center for Neuroscience, Auburn University, Alabama, USA; Alabama Advanced Imaging Consortium, Birmingham, Alabama, USA

3 – Indian Institute of Public Health, Hyderabad, Public Health Foundation of India, Hyderabad, India.

4 - Director, Diabetes Unit, KEM Hospital Research Center, India

[*] **Corresponding Author**

Dr. Rishikesh V. Behere

India Alliance Intermediate Fellow

Diabetes Unit

KEM Hospital Research Center

Pune, India

Email: rvbehere@gmail.com

Ph no: +912026061958

For peer review only

Abstract:

Introduction:

The Developmental origins of Health and Disease (DOHaD) hypothesis proposes that intrauterine and early life exposures significantly influence fetal development and risk for disease in later life. Evidence from prospective birth cohorts suggests a role for maternal B12 and folate in influencing neurocognitive outcomes in the offspring. In the Indian setting, B12 deficiency is common during pregnancy while rates of folate deficiency are lower. The long term influences of maternal nutrition during pregnancy on adult neurocognitive outcomes have not been examined. The Pune Maternal Nutrition Study (PMNS) is a pre-conceptional birth cohort into its 24th year and is considered a unique resource to study the DOHaD hypothesis. We found an association between maternal B12 status in pregnancy and child's neurocognitive status at 9 years of age. We now plan to assess neurocognitive function and MRI measurements of brain structural-functional connectivity at young adult age to study its association with maternal nutritional exposures during pregnancy.

Methods and analysis:

As part of ongoing prospective follow up in young adults of the PMNS at the Diabetes Unit, KEM Hospital Research Center, Pune India, the following measurements will be done: Neurocognitive performance (Standardized tests of Intelligence, verbal and visual memory, attention and executive functions), temperament (Adult temperament questionnaire), psychopathology (Brief symptom inventory and clinical interview on Mini Neuropsychiatric Interview 7.0). Brain Magnetic Resonance Imaging (MRI) for structural T1, resting-state functional connectivity and diffusion tensor imaging will be performed on a subset of the cohort (selected based on exposure to a lower or higher maternal B12 status at 18 weeks of pregnancy).

For peer review only

Ethics and dissemination:

The study is approved by Institutional ethics committee of KEM Hospital Research Center, Pune. The results will be shared at national and international scientific conferences and published in peer reviewed scientific journals.

Trial Registration: ClinicalTrials.gov ID: NCT03096028

Keywords: maternal nutrition, vitamin B12, neurocognitive development, brain MRI, birth cohort,

Strengths and Limitations:

1. Investigation of structural and functional neurodevelopment in the young adults in the PMNS in relation to intrauterine exposure to vitamin B12 and folate
2. A unique dataset of intergenerational life course exposures (physical growth, nutrition, childhood adversity) to investigate pathways of neurocognitive development.
3. Comprehensive behavioural measures and neuroimaging techniques (structural and functional)
4. Cross-sectional MRI measurements instead of trajectories of brain growth.
5. First life-course study in LMIC to evaluate associations of early life exposures with brain outcomes in adult age

For peer review only

Introduction:

The Developmental origins of Health and Disease (DOHaD) hypothesis (1,2) proposes that early life exposures along with post-natal environmental factors (childhood experiences, education, nutrition, infections, lifestyle, socio-economic conditions) determine risk for disease in later life. Recent investigations suggest an early life origin of neurodevelopmental disorders (autism spectrum disorders, depression, schizophrenia) (3). Early intrauterine insults can impact development of brain areas that show high neuronal growth such as hippocampus and cortical areas (4). Later life factors influence synaptic pruning and myelination (5). Altered brain structural and functional connectivity within and between hippocampal, frontal and subcortical networks is known to be associated with impaired neurocognitive functioning and neurodevelopmental disorders (6,7).

The growing fetus is wholly dependent on the mother for its nutrition. Deficiency of various micronutrients in the mother during critical phases of foetal development can affect organ structure and function, hence programming the risk for chronic disease in later life (fetal programming) (8). Recently there is considerable interest in the role of maternal vitamin B12 and folate in fetal programming.

Vitamin B12 is important for nerve myelination and its deficiency is associated with subacute combined degeneration of spinal cord, depression and dementia(9). The role of folate deficiency in risk for neural tube defects is well known. Both these vitamins influence the one carbon metabolism cycle(10).

Observations from large prospective cohort studies such as the Norwegian mother and child birth cohort (MoBa) (11) and Generation R support association of poor maternal folate status with poorer neurocognitive functioning, greater childhood behavioural problems(12–14), smaller brain volumes(15) and risk for autism(16,17). Prospective cohort studies from India support the associations of lower maternal vitamin B12 or folate with higher risk for NTD and poorer cognitive functions in offspring in early (30-48 months) and late (9-10 years) childhood. Systematic reviews support a high level of evidence for association of low maternal

For peer review only - http://bmjopen.bmj.com/site/about/guidelines.xhtml

vitamin B12 with high risk for NTD and moderate level evidence for poor cognitive outcomes in the offspring (18,19). It must be noted that these observational studies do not support causality. Causality can be examined by randomized controlled trials; which need time and involve ethical considerations. Techniques such as Mendelian Randomization allow testing for causality of observed associations(20). The Avon Longitudinal Study on Parents And Children (ALSPAC) reported association between low maternal dietary intake of vitamin B12 and lower IQ scores at 8 year and genetic determinants of vitamin B12 (FUT2gene) using this technique (21).

In India, vitamin B12 deficiency is widely prevalent in women in the reproductive age group while rates of folate deficiency are low (22–24). The vitamin B12 deficiency is attributable to practice of vegetarianism and low socio-economic status. Public health policy mandates only iron-folic acid supplementation in adolescents and pregnant women. It is also a common clinical practice to supplement high dose folic acid (5mg) to pregnant women to prevent Neural Tube Defects (NTD). This scenario inadvertently promotes an imbalance between vitamin B12 and folate status during pregnancy(19,25) which could enhance risk of adverse health outcomes in the offspring including aberrant neurodevelopment. In the presence of vitamin B12 deficiency, folate is trapped in its unusable methyl form and methyl groups are unavailable for further biochemical reactions. This interferes with important cell processes such as purine pyrimidine synthesis, DNA repair, and epigenetic regulation (DNA methylation). Unavailability of methyl groups prevents conversion of homocysteine to S-Adenosyl Methionine leading to hyper-homocysteinemia which can adversely affect vascular endothelial and placental function. Indiscriminate supplementation in the presence of vitamin B12 deficiency leads to accumulation of unmetabolized folic acid (UMFA) which are known to have neurotoxic effects(26,25). Animal experiments performed in India, have examined the role of a maternal vitamin B12-folate micronutrient imbalance and show that offspring dams of mothers fed on a low vitamin B12-highfolate diet have poorer spatial memory functions, lower brain hippocampal weight, reduced neurotrophic factor BDNF and reduced TRK gene expression(27,28). However, no study in India has examined long term effects of maternal

For peer review only - http://bmjopen.bmj.com/site/about/guidelines.xhtml

vitamin and folate during pregnancy on offspring neurodevelopmental outcomes in young adulthood.

The Pune Maternal Nutrition Study (PMNS) is a pre-conceptional birth cohort with serial data on maternal nutritional status, birth outcomes and offspring nutrition, physical growth and development. We have observed high prevalence of maternal vitamin B12 deficiency during pregnancy in our cohort. We have described markers of low vitamin B12 status in the mother (high homocysteine) to be causally associated with low birth weight in the offspring(29). A low vitamin B12-high folate dietary pattern was associated with adverse metabolic outcomes in the offspring at 6 years(24). We have earlier demonstrated in this cohort that exposure to a low maternal vitamin B12 was associated with poor cognitive functioning in the offspring at 9 years of age(30). The offspring of this cohort are now in their early 20's and this provides a unique opportunity to examine the long-term effects of maternal nutrition status during pregnancy on neurocognitive outcomes in young adulthood.

Aims and objectives:

The key aim of the study is to examine whether maternal micronutrient status during pregnancy (B12, folate) is associated with neurocognitive performance, structural and functional connectivity and risk for common mental disorders in young adult offspring of the PMNS birth cohort.

Primary objectives:

1. To examine associations between maternal vitamin B12, folate, and their interaction at 18 and 28 weeks of pregnancy with neurocognitive outcomes (neuropsychological test performance) and risk for common mental disorders (temperament, presence of depression or anxiety disorders) in young adulthood; controlling for the effects of other

For peer review only - http://bmjopen.bmj.com/site/about/guidelines.xhtml

nutrients (calorie, protein and fat intake, and levels of vitamin C,D, ferritin) and later life developmental influences (education, adverse childhood experiences, offspring nutrition, physical growth and development, metabolic status).

2. To examine the association of maternal nutritional status during pregnancy with brain structure (grey matter volume and cortical thickness), and structural/functional connectivity (using Diffusion Tensor Imaging – DTI and resting state functional Magnetic Resonance Imaging – rsfMRI, respectively).
3. To test the causality of association between maternal vitamin B12, folate, and neurocognitive outcomes using maternal and offspring genetic determinants of vitamin B12 (FUT2, FUT6, TCN2).

Hypotheses:

We hypothesize that a lower maternal vitamin B12, higher folate, higher homocysteine nutrient pattern during pregnancy adversely impacts development of brain regions. This will be reflected in brain imaging as reduced brain volume as measured through anatomical images, weaker connections in structural/functional networks (hippocampal and frontal-subcortical connections) involved in cognitive processing (memory, executive functions) as measured through DTI and resting state fMRI, respectively. These structural and functional changes will in turn result in poorer neurodevelopmental outcomes (lower neuropsychological test performance, adverse temperamental traits and higher risk for common mental disorders) in the young adult offspring."

Methods and Analysis:

Study setting:

Pune Maternal Nutrition Study:

For peer review only

The PMNS is a pre-conceptional cohort, established in 6 villages of Pune district, Maharashtra state, India in 1993 in collaboration with Dr DJP Barker and Dr Caroline Fall and is considered a unique resource in the world to examine the DOHaD paradigm. Initially, 2466 women (F0 generation) in the reproductive age group participated in the study. 814 women who became pregnant in the period June 1994 to April 1996 were enrolled for the study. Of these 797 pregnancies (with singleton pregnancy and period of gestation less than 21 weeks) were included. 762 live births occurred (F1 generation). This cohort has data on pre-conceptional parental size, maternal nutrition and size at birth of the offspring. Maternal micronutrients vitamin B12, red cell folate and ferritin have been measured from blood samples collected at 18 and 28 weeks of gestation. Vitamin B12 levels were measured using the microbiological assay technique and red cell folate and ferritin by radioimmunoassay(24,31). Children are being regularly followed up for growth and development (body size and DXA body composition), nutrition, and cardio-metabolic risk factors (6,12&18 years of age). Biological samples have been collected and stored in a bio-repository and are available for assessments. The other strengths of this cohort are good community participation and follow up rates of over 90% over the years. At 18 years follow up, 690 of the original 762 live births in the cohort were followed up.

The participants (F1 generation) of the cohort are also part of a randomized controlled trial (Pune Rural Intervention in Young Adolescents, PRIYA) (32) to examine intergenerational effects of micronutrient supplementation on various health outcomes. In 2012 at the age of 17 years 557 eligible members were randomized as part of the nutrition intervention trial into one of 3 groups 1) 2 micrograms/day of vitamin B12, 2) 2 micrograms/day of Vitamin B12 with UNIMAPP multivitamins and 20g milk powder 3) placebo. The trial was completed in the boys in December 2017 and in the girls in September 2019. The effects of later life intervention will be adjusted during statistical analysis. (Figure 1 – Depicts time line of measurements in the PMNS cohort).

For peer review only - http://bmjopen.bmj.com/site/about/guidelines.xhtml

Subject selection:

All subjects of the PMNS cohort (n=690) will be invited for participation in the study (Figure 2) subject to following inclusion criteria: 1) Maternal vitamin B12, folate levels measured during pregnancy 2) Serial measurements done at 6,12 and 18 years of age. Neurocognitive assessments, temperament and mental health assessments will be performed in all consenting subjects. Brain MRI will be performed on a subset of the cohort selected based on exposure to maternal vitamin B12 levels at 18 weeks of gestation. Median vitamin B12 level at 18-week gestation was 153pM (103,175 pM being the 1st and 4th quartiles). Description of maternal characteristics at 18 and 28-week gestation and offspring birth characteristics are provided in the supplementary material - 1. Of the 690 participants still in follow up, 152 subjects (78 males, 74 females) whose mothers had plasma vitamin B12 concentration <103pM and 154 subjects (88 males, 66 females) whose mothers had plasma vitamin B12 concentration >175pM will be eligible for participation in the brain MRI scans. 100 participants each from these two groups (total of 200 subjects) will be randomly selected..

Assessments:

The exposures and outcome measurements are summarised in Table – 1.

Behavioural assessments:

These include 1) Standardized neurocognitive test batteries - Wechsler's adult intelligence scale (WAIS-IV), Wechsler Memory Scale (WMS), Auditory verbal learning test (AVLT), Color trail test. 2) Adult Temperament Questionnaire (ATQ) 3) Mental health assessments - Brief symptom inventory (BSI), Mini Neuropsychiatric Interview (MINI plus 7.0). Further details of behavioural assessments are provided in the supplementary material-2. 4) Early life stress. - WHO Adverse Childhood Experiences International Questionnaire (ACE-IQ).

For peer review only - http://bmjopen.bmj.com/site/about/guidelines.xhtml

	Exposures	Tests	Variables measured
**1.**	Maternal Micronutrient status and homocysteine at 18 and 28 wks pregnancy	Biochemical assays	Vitamin B12, red cell folate, homocysteine
	**Outcomes**	**Tests**	**Variables measured**
1.	Neurocognitive performance	WAIS-IV<br>WMS<br>AVLT<br>Color Trails test	Verbal comprehension, perceptual reasoning, working memory, processing speed, Full scale IQ.<br>Immediate Memory, delayed memory and working memory.<br>Errors in Immediate and delayed recall<br>Time taken and errors for Color trails test A & B.
2.	Temperament	Adult Temperament Questionnaire	Negative affect, extraversion, orienting sensitivity, effortful control
3.	Common mental disorders	Brief Symptom Inventory<br>Mini Neuropsychiatric Interview 7.0 for DSM 5 diagnosis	Global severity index<br>DSM5 diagnosis of past or current Major Depressive disorder, suicidality, Panic disorder, Agoraphobia, Social anxiety disorder, generalised anxiety disorder
5.	Brain Imaging	Structural T1 MPRAGE<br>Resting state fMRI<br>Diffusion tensor imaging	Cortical volume and thickness<br>Resting state functional connectivity<br>Tractography, FA and MD values in relevant white matter tracts
	**Life-course variables**		
	Birth		Birth weight, length, head circumference, sum of skin folds
	Later life growth and development		Height, BMI, head circumference sum of skin folds at 6, 12, 18 years
	Later life nutrition		Hemoglobin, vitamin B12, folate, homocysteine, ferritin, glucose, lipids at 6,12,18 years
	Socio-environmental		Mothers education, fathers education, standard of living index, adverse childhood experiences

For peer review only

Table -1 Exposures and Outcomes defined in the study

Magnetic resonance imaging:

Anatomical data using structural 3D T1 weighted MPRAGE (magnetization prepared rapid gradient echo) sequence, resting state fMRI (rs-fmri) with 2D gradient-echo EPI (echo planar imaging) sequence, and Diffusion Tensor Imaging (DTI) using RESOLVE sequence will be acquired on a 3 Tesla Siemens Skyra scanner.

Image acquisition:

For structural imaging, 3D T1-weighted MPRAGE images will be acquired (TR=2300 ms, TE=2.26 ms, TI=900 ms, Flip angle=8 degrees, slice thickness=1 mm, in-plane resolution= 1×1 mm^2, GRAPPA parallel imaging with acceleration factor of 2, 192 slices, FOV readout=256 mm, FOV phase encoding= 100%, and acquisition time of 5 min and 21 s).

For functional imaging, T2*-weighted echo-planar imaging sequence will be used to acquire BOLD fMRI data while participants are resting with eyes open and fixated, and not thinking about anything specific. A resting state fMRI scan helps to characterize brain-wide networks from a single scan as opposed to task-based fMRI which characterizes evoked responses in specific brain systems. Thirty six axial slices will be acquired covering the whole brain with a slice thickness of 3 mm, in-plane resolution of 3×3 mm^2, TR=2000 ms; TE=30 ms; flip angle=90^0, FOV readout=256 mm, FOV phase encoding= 100%, GRAPPA parallel imaging with acceleration factor of 2, and 300 measurements with an acquisition time of 10 min and 8 s. PACE (prospective acquisition correction) motion correction will be employed in this sequence. (33,34)

For peer review only - http://bmjopen.bmj.com/site/about/guidelines.xhtml

Diffusion-weighted images will be acquired using the RESOLVE (readout segmentation of long variable echo-trains) sequence given its robustness against susceptibility artifacts. Scan parameters for RESOLVE will be: Thirty-six axial slices covering the whole brain with a slice thickness of 3 mm, in-plane resolution of 1.4×1.4 mm^2, TR=5460 ms; TE=73/109 ms; flip angle=180^0, FOV readout=220 mm, FOV phase encoding= 100%, GRAPPA parallel imaging with acceleration factor of 2, 30 diffusion directions, two diffusion weightings (b-values) of 0 and 1000 s/mm^2 and an acquisition time of 14 min and 19 s.

All MRI images will be visually inspected for quality assessment and reviewed by a radiologist to rule out any structural abnormalities.

Data management:

Data will be stored at the Diabetes Unit, KEM Hospital Research Center, Pune, India. Data collected will be first entered in a paper clinical record form and double entered into an open clinical platform on a computer. Data is downloaded and checked for extreme or missing values. Data on maternal micronutrient status during pregnancy (nutrition intake, circulating micronutrients) and intermediate variables (birth size, childhood nutrition, growth and development, metabolic factors (insulin resistance, glycemic status, adiposity), socio demographic variables and socio-economic status have been assessed previously on the cohort. These will be extracted and used for analysis.

Statistical methods:

Power estimation:

There are 690 members of the cohort available for assessments. Assuming a 80% participation rate (from experience of previous studies on the cohort and accounting for

For peer review only - http://bmjopen.bmj.com/site/about/guidelines.xhtml

migration) we estimate that 550 subjects would be recruited for the study. Using a test at 5% significance level, the study will have 80% power to detect an association of 0.11 standard deviation of continuous outcome (eg. neurocognitive test score) per standard deviation of continuous exposure (eg. maternal vitaminB12 level). Study of 200 subjects (100 in each group) for Brain imaging will have 80% power for an effect size 0.3 and significance at p <0.05 for detecting difference between the two maternal vitamin B12 exposure groups.

Current Status and impact of COVID-19 pandemic:

The study protocol was approved for funding in 2017. Subject recruitment was initiated in May 2018 and had to be halted in March 2020 due to the COVID-19 pandemic. As of March 2020 neurocognitive assessments have been performed on 386 participants of the cohort out of intended 550 participants and Brain MRI scans obtained on 195 participants our of intended 200. We will try to recruit more participants once the situation is conducive. We acknowledge that due to the COVID-19 pandemic there will be some reduction in the power of the study. The results will be reported as per emerging guidelines on reporting clinical studies affected by the COVID-19 pandemic(35) .

Plan of Data analysis:

Objective 1: This examines the association between intrauterine nutritional exposures and neurocognitive outcomes in young adult age:

Our primary exposure of interest is maternal vitamin B12 and red cell folate levels. Due to lack of reliable clinical cutoffs for these micronutrients during pregnancy for our population, we prefer to use the distribution within population to classify participants. Such an approach has been used earlier to report metabolic outcomes in the offspring in this cohort (24). Distribution of outcome variables such as neurocognitive performance scores will be visualized by using

For peer review only - http://bmjopen.bmj.com/site/about/guidelines.xhtml

different plotting techniques such as box plot and scatter plot. Univariate analysis (Pearson' correlation and linear regression) will be performed to examine the relationships between the primary exposures, life course variables and neurocognitive outcomes (table -1). We expect to construct multivariate linear (if outcome is continuous) or logistic regression (categorical outcome) models to examine associations of our primary exposures with neurocognitive outcomes and extend the models to examine potential modifying and mediating effects of life-course variables on the associations. Finally, we will adjust for the vitamin B12 intervention status (vitamin B12 intervention received yes or no) in the PRIYA trial. We will follow this broad scheme of analysis. Depending on observed univariate associations we may construct additional multivariate models.

Given the multitude of factors and possible collinearity we will use dimensionality reduction techniques (such as Principal Component Analysis - PCA). The components might be used to construct path analytical models for exploring associations between outcomes.

Objective 2: This examines the association between maternal nutritional exposures and brain structural functional connectivity:

1. Structural Brain MRI analysis
    a. Voxel-based Morphometry on T1 MPRAGE anatomical images will be performed using statistical parametric mapping in Matlab. Pre-processing steps will involve co-registration, segmentation. Spatial normalisation will transform all subject data to the same stereotactic space. Gray matter images will be smoothed by convolving with an isotropic Gaussian kernel. Statistical analysis using General Linear Model will be used to identify regions of grey matter concentration that are significantly associated with our exposures of interest.

For peer review only - http://bmjopen.bmj.com/site/about/guidelines.xhtml

b. Cortical volume, thickness and hippocampal segmentation: A surface based morphometric technique will be employed to measure cortical volume and thickness. We will use freesurfer software for image analysis. Standard steps include intensity normalisation, skull stripping and removal of non-brain tissues followed by segmentation, topology correction, registration and automated parcellation. Cortical volume and thickness will be computed for 68 regions (34 in each hemisphere) using the Desikan-Kelliany Atlas. The hippocampal subfield segmentation tool in freesurfer will be employed to compute volumes of the various hippocampal structures as per the inbuilt atlas which defines 11 hippocampal subfield structures.

2. Structural Connectivity – Diffusion tractography analysis: Diffusion weighted images will be analysed using the "The Diffusion Toolbox" software tool in the FMRIB Software Library (FSL; http://www.fmrib.ox.ac.uk/fsl/fdt/index.html). After pre-processing including motion correction and eddy current correction, the DTI indices fractional anisotropy (FA), mean diffusivity (MD) and radial diffusivity (RD) will be computed. Structural connectome generation will be performed on the MRItrix3 software. Pre-processing steps will involve denoising and correction for eddy currents. The b0 volume of pre-processed images will be extracted and registered to the T1 image using FSL's BET tool. The fiber orientation dispersion functions (FODs) will be generated for white matter and normalised. Probabilistic streamlines tractography will be performed by dynamically seeding the normalized white matter FOD's. The whole brain tractogram thus generated will be used for structural connectome generation.

3. Functional Connectivity: CONN software (https://web.conn-toolbox.org/) will be used for the analysis of rs-fMRI data. This will involve standard pre-processing steps

For peer review only - http://bmjopen.bmj.com/site/about/guidelines.xhtml

such as realignment, normalization to MNI space, elimination of temporal linear trends and regressing out nuisance covariates such as residual motion and physiological artifacts. *aCompCor* will be used for minimizing physiological artifacts. Motion censoring and interpolation will also be used to remove the effects of large frame wise displacements due to head motion. Following pre-processing, mean time series will be extracted from 91 cortical and 15 sub-cortical regions in the FSL Harvard-Oxford atlas, as well as from 26 cerebellar regions in the AAL atlas. These time series will be used in a whole brain functional connectivity analysis employing Pearson's correlation coefficient between the mean time series.

For each modality, the following statistical comparisons will be performed. We will first perform correlations between maternal vitamin B12 and folate exposures with brain MRI outcomes to examine for any linear relationships. We will use an analysis of covariance (ANCOVA) model to examine differences in MRI outcomes between low and high maternal vitamin B12 groups. Similarly, we will examine for differences between low and high maternal red cell folate groups (selected based on median value of maternal red cell folate) and for any interaction between maternal vitamin B12 and folate status. Variance due to subjects' age, gender, total intracranial volume, adverse childhood experiences, will be regressed out of the analyses. Effects of additional factors (such as current vitamin B12 status of offspring, education status, socio-economic status) will be examined.

For making inferences from multimodal imaging data, we will adopt a hierarchical approach. Accordingly, we will perform voxel-wise tests for difference in grey matter volume (or cortical thickness). For brain regions which are significant, DTI-based structural connectivity and fMRI-based functional connectivity between those regions will be investigated. This strategy assures that the effects of interest have both structural and functional etiologies, and hence are likely to be robust and reproducible.

For peer review only - http://bmjopen.bmj.com/site/about/guidelines.xhtml

Objective 3:

This examines causality of association between maternal nutritional factors and neurocognitive outcomes. The genetic determinants of vitamin B12 have been described in our population using GWAS (36) which will be used to calculate a gene risk score of vitamin B12 deficiency. Mendelian randomization analysis (triangulation and instrumental variable analysis) using the genetic risk score for vitaminB12 deficiency and homocysteine (MTHFRrs1801133) will be performed.

Patient and public involvement:

The present study is a follow up of participants of the Pune Maternal Nutrition Study birth cohort. This cohort was set up in 1993 from six villages within Pune district in India. The cohort has been serially evaluated every 6 years (at 6,12,18 years) and is currently in the 24th year of its follow up. The cohort participants were also part of randomized controlled trial from 2012-2019. We are also following up the children born in the next generation of the cohort. The cohort has a high follow up rate of 90% over the last 18 years. This was possible due to active community engagement and involvement of social workers who are from the same community and interact with cohort participants at regularly. Information on results of our studies are shared with cohort participants through public engagement initiatives.

Ethics and dissemination:

The study will be carried out as per the ethical guidelines provided by the Indian Council of Medical Research. The study has been approved by the Institutional Ethics Committee of the KEM Hospital Research Center, Pune (KEMHRC/RVM/EC/1249). Study is registered with clinicaltrials.gov (ID-NCT 03096028). The cohort participants will be approached by the field workers who have been following them in the community for last many years. Participants will

For peer review only - http://bmjopen.bmj.com/site/about/guidelines.xhtml

be provided the study information sheet and an opportunity to discuss their doubts and concerns regarding the study with the investigator and then recruited after they sign a written informed consent.

The data generated from this study will be shared with the scientific community at local, national and international conferences, and published in open access peer reviewed journals. The major findings will be shared with the general public, study participants and their families in lay terms through public engagement initiatives.

The study will help build a database on neurocognitive and mental health outcomes and brain imaging in young adulthood in a pre-conceptional birth cohort. The results of the association of maternal vitamin B12 during pregnancy and long term neurodevelopmental outcomes will inform public health actions for nutritional health of adolescent girls and women of reproductive age.

Figure 1 - Understanding neurocognitive development using a life course approach in the PMNS

Figure 2 - Selection of subjects from the PMNS cohort for the study

Competing interests:

The authors declare that they have no competing interests.

For peer review only - http://bmjopen.bmj.com/site/about/guidelines.xhtml

Funding:

This work is supported by the DBT/Wellcome Trust India Alliance Fellowship [IA/CPHI/161502665] awarded to Dr Rishikesh V. Behere.

Author's contribution:

RVB and CSY conceptualised and designed the study and wrote the manuscript. SB designed the statistical analysis plan and power estimation. GD contributed to the brain imaging methods and analysis and framing the study objectives. All authors read and approved the final manuscript.

References:

1. Hales CN, Barker DJ. The thrifty phenotype hypothesis. Br Med Bull. 2001;60–5.

2. Fleming TP, Watkins AJ, Velazquez MA, Mathers JC, Prentice AM, Stephenson J, et al. Origins of lifetime health around the time of conception: causes and consequences. Lancet Lond Engl. 2018 May 5;391(10132):1842–52.

3. Schlotz W, Phillips DI. Fetal origins of mental health: evidence and mechanisms. Brain Behav Immun. 2009;23(7):905–16.

4. Gilchrist C, Cumberland A, Walker D, Tolcos M. Intrauterine growth restriction and development of the hippocampus: implications for learning and memory in children and adolescents. Lancet Child Adolesc Health. 2018 Oct;2(10):755–64.

5. Langen CD, Muetzel R, Blanken L, van der Lugt A, Tiemeier H, Verhulst F, et al. Differential patterns of age-related cortical and subcortical functional connectivity in 6-to-10 year old children: A connectome-wide association study. Brain Behav. 2018 Aug;8(8):e01031.

6. Liu J, Chen Y, Stephens R, Cornea E, Goldman B, Gilmore JH, et al. Hippocampal functional connectivity development during the first two years indexes 4-year working memory performance. Cortex. 2021 May;138:165–77.

7. Yoon YB, Shin W-G, Lee TY, Hur J-W, Cho KIK, Sohn WS, et al. Brain Structural Networks Associated with Intelligence and Visuomotor Ability. Sci Rep. 2017 Dec;7(1):2177.

8. Yajnik CS, Deshmukh US. Maternal nutrition, intrauterine programming and consequential risks in the offspring. Rev Endocr Metab Disord. 2008 Sep;9(3):203–11.

For peer review only

9. Misra UK, Kalita J, Das A. Vitamin B12 deficiency neurological syndromes: a clinical, MRI and electrodiagnostic study. Electromyogr Clin Neurophysiol. 2003 Feb;43(1):57–64.

10. Yajnik CS, Deshmukh US. Fetal programming: maternal nutrition and role of one-carbon metabolism. Rev Endocr Metab Disord. 2012 Jun;13(2):121–7.

11. Magnus P. The Norwegian Mother and Child Cohort Study (MoBa)–new research possibilities. Vol. 17. Norsk epidemiologi; 2007.

12. Jacka FN, Ystrom E, Brantsaeter AL, Karevold E, Roth C, Haugen M, et al. Maternal and early postnatal nutrition and mental health of offspring by age 5 years: a prospective cohort study. J Am Acad Child Adolesc Psychiatry. 2013;52–10.

13. Roza SJ, van Batenburg-Eddes T, Steegers EA, Jaddoe VW, Mackenbach JP, Hofman A, et al. Maternal folic acid supplement use in early pregnancy and child behavioural problems: the Generation R Study. Br J Nutr. 2010;103–03.

14. Schlotz W, Jones A, Phillips DI, Gale CR, Robinson SM, Godfrey KM. Lower maternal folate status in early pregnancy is associated with childhood hyperactivity and peer problems in offspring. J Child Psychol Psychiatry. 2010;51–5.

15. Ars CL, Nijs IM, Marroun HE, Muetzel R, Schmidt M, Steenweg-de Graaff J, et al. Prenatal folate, homocysteine and vitamin B12 levels and child brain volumes, cognitive development and psychological functioning: the Generation R Study. Br J Nutr. 2016 Jan 22;1–9.

16. Suren P, Roth C, Bresnahan M, Haugen M, Hornig M, Hirtz D, et al. Association between maternal use of folic acid supplements and risk of autism spectrum disorders in children. JAMA. 2013;309–6.

17. Steenweg-de Graaff J, Ghassabian A, Jaddoe VW, Tiemeier H, Roza SJ. Folate concentrations during pregnancy and autistic traits in the offspring. The Generation R Study. Eur J Public Health. 2015;25–3.

18. Veena SR, Gale CR, Krishnaveni GV, Kehoe SH, Srinivasan K, Fall CH. Association between maternal nutritional status in pregnancy and offspring cognitive function during childhood and adolescence; a systematic review. BMC Pregnancy Childbirth. 2016 Aug 12;16:220.

19. Behere RV, Deshmukh AS, Otiv S, Gupte MD, Yajnik CS. Maternal Vitamin B12 Status During Pregnancy and Its Association With Outcomes of Pregnancy and Health of the Offspring: A Systematic Review and Implications for Policy in India. Front Endocrinol. 2021 Apr 12;12:619176.

20. Smith GD, Ebrahim S. “Mendelian randomization”: can genetic epidemiology contribute to understanding environmental determinants of disease? Int J Epidemiol. 2003 Feb;32(1):1–22.

21. Bonilla C, Lawlor DA, Taylor AE, Gunnell DJ, Ben-Shlomo Y, Ness AR, et al. Vitamin B-12 status during pregnancy and child’s IQ at age 8: a Mendelian randomization study in the Avon longitudinal study of parents and children. PloS One. 2012;7(12):e51084.

For peer review only

22. Samuel TM, Duggan C, Thomas T, Bosch R, Rajendran R, Virtanen SM, et al. Vitamin B(12) intake and status in early pregnancy among urban South Indian women. Ann Nutr Metab. 2013;62(2):113–22.

23. Pathak P, Kapil U, Yajnik CS, Kapoor SK, Dwivedi SN, Singh R. Iron, folate, and vitamin B12 stores among pregnant women in a rural area of Haryana State, India. Food Nutr Bull. 2007 Dec;28(4):435–8.

24. Yajnik CS, Deshpande SS, Jackson AA, Refsum H, Rao S, Fisher DJ, et al. Vitamin B12 and folate concentrations during pregnancy and insulin resistance in the offspring: the Pune Maternal Nutrition Study. Diabetologia. 2008 Jan;51(1):29–38.

25. Behere RV, Yajnik CS. Low vitamin B-12–high folate status in adolescents and pregnant women may have deleterious effects on health of the offspring. Am J Clin Nutr. 2021 Apr 6;113(4):1057–9.

26. Bailey R, Jun S, Murphy L, Green R, Miller J. High Folate and Vitamin Low B12 Status: Potential Interactions with Cognitive Function Among U.S. Older Adults, NHANES 2011–2014. Curr Dev Nutr. 2020 Jun 1;4(Supplement_2):1189–1189.

27. Sable P, Dangat K, Kale A, Joshi S. Altered brain neurotrophins at birth: consequence of imbalance in maternal folic acid and vitamin B(1)(2) metabolism. Neuroscience. 2011;190–127.

28. Sable P, Kale A, Joshi A, Joshi S. Maternal micronutrient imbalance alters gene expression of BDNF, NGF, TrkB and CREB in the offspring brain at an adult age. Int J Dev Neurosci. 2014;34–24.

29. Yajnik CS, Chandak GR, Joglekar C, Katre P, Bhat DS, Singh SN, et al. Maternal homocysteine in pregnancy and offspring birthweight: epidemiological associations and Mendelian randomization analysis. Int J Epidemiol. 2014 Oct;43(5):1487–97.

30. Bhate V, Deshpande S, Bhat D, Joshi N, Ladkat R, Watve S, et al. Vitamin B12 status of pregnant Indian women and cognitive function in their 9-year-old children. Food Nutr Bull. 2008 Dec;29(4):249–54.

31. Rao S, Yajnik CS, Kanade A, Fall CH, Margetts BM, Jackson AA, et al. Intake of micronutrient-rich foods in rural Indian mothers is associated with the size of their babies at birth: Pune Maternal Nutrition Study. J Nutr. 2001 Apr;131(4):1217–24.

32. Kumaran K, Yajnik P, Lubree H, Joglekar C, Bhat D, Katre P, et al. The Pune Rural Intervention in Young Adolescents (PRIYA) study: design and methods of a randomised controlled trial. BMC Nutr [Internet]. 2017 Dec [cited 2019 Jan 22];3(1). Available from: http://bmcnutr.biomedcentral.com/articles/10.1186/s40795-017-0143-5

33. Lanka P, Deshpande G. Combining Prospective Acquisition CorrEction (PACE) with retrospective correction to reduce motion artifacts in resting state fMRI data. Brain Behav. 2019;9(8):e01341.

34. Lanka P, Deshpande G. Resting state fMRI data from subjects scanned with the EPI-PACE (Echoplanar Imaging–Prospective Acquisition CorrEction) sequence. Data Brief. 2018;20:2072–5.

For peer review only

35. Perlis RH, Haneuse SJPA, Rubenfeld GD, Fihn SD, Rivara FP. Reporting Clinical Studies Affected by the COVID-19 Pandemic: Guidelines for Authors. JAMA Netw Open. 2021 Jan 14;4(1):e2036155.

36. Nongmaithem SS, Joglekar CV, Krishnaveni GV, Sahariah SA, Ahmad M, Ramachandran S, et al. GWAS identifies population-specific new regulatory variants in FUT6 associated with plasma B12 concentrations in Indians. Hum Mol Genet. 2017 01;26(13):2589.

For peer review only

For peer review only - http://bmjopen.bmj.com/site/about/guidelines.xhtml

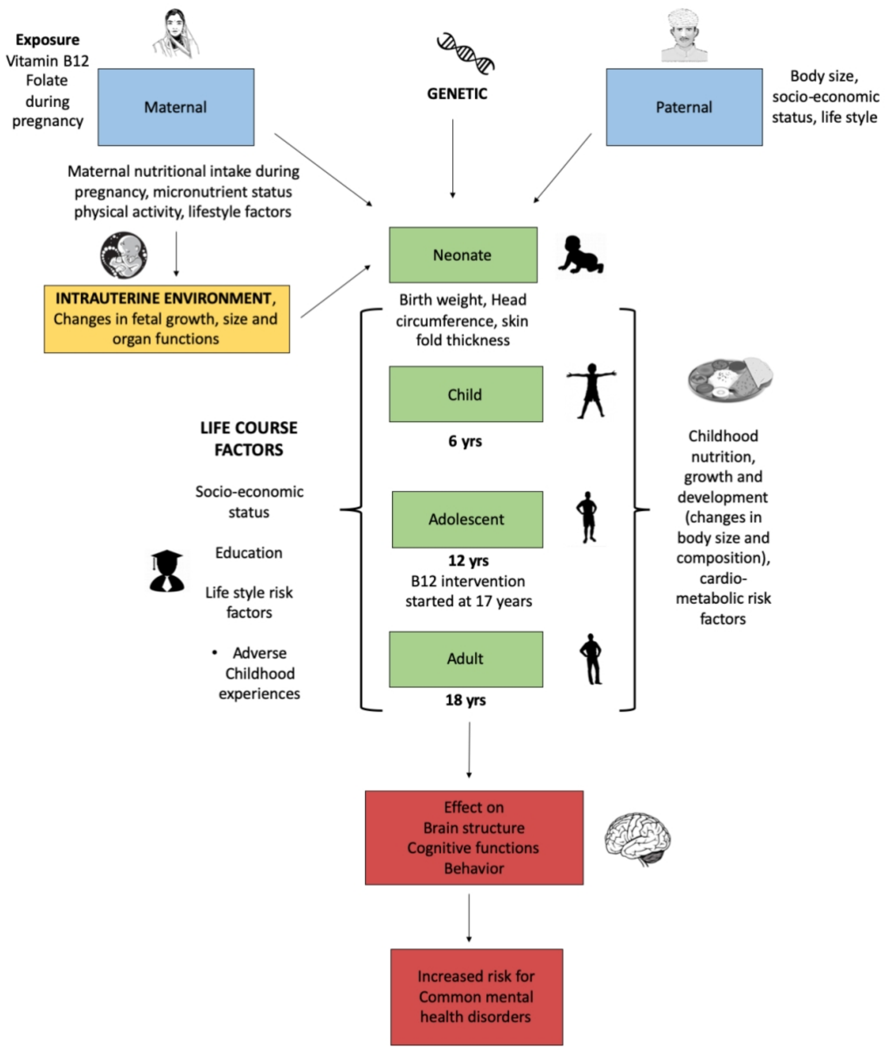

Figure 1 - Understanding neurocognitive development using a life course approach in the PMNS

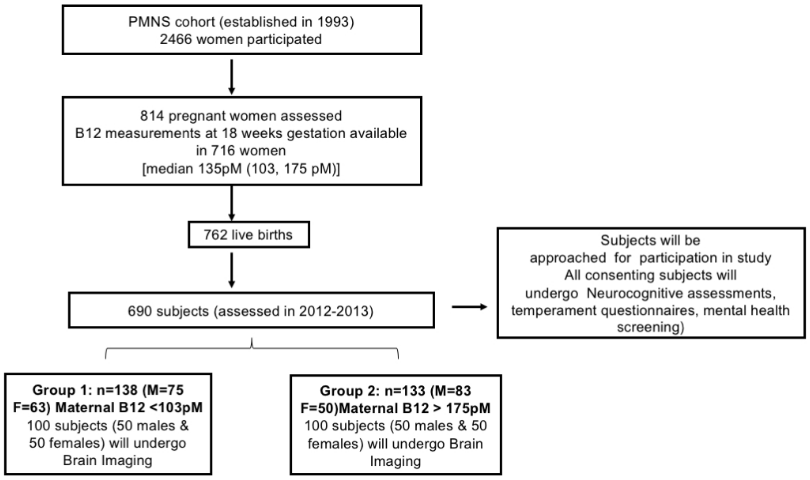

Figure 2 - Selection of subjects from the PMNS cohort for the study

	n	Median (Q1, Q3)
**Maternal nutritional variables at 18 wks pregnancy**		
Total calorie intake (kcal)	796	1736.5 (1402.4,2074.3)
Plasma B12 (pmol/L)	716	135.1 (103.4,175.3)
Red cell folate (ng/mL)	698	388.0 (306,490.3)
Plasma Homocysteine (micromol/L)	717	8.2 (6.8,10.4)
Serum Ferritin (microgm/L)	745	13.0 (8,23)
**Maternal nutritional variables at 28 wks pregnancy**		
Total calorie intake (kcal)	749	1623.8 (1318.8,1974.8)
Plasma B12 (pmol/L)	656	121.5 (93.5 , 156.9)
Red cell folate (ng/mL)	617	421.0 (326,556.5)
Plasma Homocysteine (micromol/L)	653	8.6 (6.7,10.9)
Serum Ferritin (microgm/L)	651	11.0 (7, 20)

For peer review only

**Birth characteristics**		
Birth weight adjusted to 40 wks (gms)	718	2722.2 (2509.8,2948.0)
Length at birth adjusted to 40 wks (cms)	708	48.2 (47.0,49.3)
Head circ at birth adjusted to 40 wks	709	33.4 (32.5,34.2)

Supplementary material – 1 Maternal characteristics at 18 and 28 week of pregnancy and birth characteristics of the PMNS cohort

For peer review only

Supplementary material - 2.

Details of behavioural assessment of neurocognitive outcomes

a. Neurocognitive assessment:

Standardized battery of neuropsychological tests to assess Intelligence and the domains of executive functions, working memory, and verbal memory.

1. Wechsler's adult intelligence scale (WAIS-IV): Wechsler Adult Intelligence scale (36) (WAIS)-4th edition measures cognitive ability in adults between the ages of 16 years 0 months and 84 years 11 months. The test provides information on the subject's performance on 10 subtests, 4 sub scales i.e. Verbal Comprehension Index scale (VCI), Perceptual Reasoning Index scale (PRI), Working Memory Index scale (WMI) and Processing Speed Index scale (PSI), and gives a final measure of Full-scale IQ (FSIQ).
2. Wechsler's Memory Scale: Wechsler Memory Scale (WMS) (37) measures memory functioning in adults between the ages of 16 years and 90 years. WMS is comprised of eleven sub-tests, and the person's performance is reported on six indices. In this study, six sub-tests will be administered to measure performance on visual immediate memory, visual delayed memory and working memory.
3. Auditory verbal learning test: This is a test of verbal learning and memory (38). The individual is read out a list of 15 words and is asked to recall the words. The list (list A) is initially read out 5 times and recall is tested after each reading. After this, a second list is read out (list B) and recall is tested. Following the recall of list B, recall of list A is immediately tested again. After a 20-minute gap, the delayed recall of list A is tested. The individual is scored based on number of correct answers, omissions, commissions, and total errors.

For peer review only

4. Color trail test: This is a test of executive functions. It involves of 2 parts A & B where subjects have to connect numbered circles from 1 to 25 followed by connecting numbered circles in alternating colors (yellow and pink). Time to complete task and number of errors are recorded. Longer time indicates poor performance.

b. Psychological assessments:

Adult Temperament Questionnaire (ATQ): This 60-item self-report questionnaire is part of Rothbart's scales of temperament. It Yields scores on the domains of negative affect, effortful control, extraversion and orienting sensitivity

c. Mental Health assessment

Brief symptom inventory (BSI) – This is a self or interviewer administered questionnaire that evaluates psychological distress and psychiatric disorders in people. It consists of 53 items covering nine symptom dimensions. Participants rate their feelings about a given item on a 5-point rating scale ranging from 0 (not at all) to 4 (extremely). Rankings characterize the intensity of distress during the past seven days. The test yields score on 9 psychopathology domains and three global indices of distress: Global Severity Index, Positive Symptom Distress Index, and Positive Symptom Total.

MINI (Mini Neuropsychiatric interview plus 7.0): All subjects will be assessed on a structured clinical interview (39) which yields syndromal psychiatric diagnosis based on the DSM-5**.** CMD of interest assessed would be depression and anxiety disorders. All subjects meeting criteria on the MINI will be reassessed by comprehensive clinical mental status examination to confirm the diagnosis. Any subject found to have syndromal psychiatric diagnosis will be offered treatment as per standard clinical protocols.

d. Early life stress: WHO Adverse Childhood Experiences International Questionnaire (ACE-IQ) is a subject rated questionnaire to assess ACE upto age 18 years. Responses are coded

For peer review only

into binary scores which are summated to yield a total ACE-IQ score. A higher score indicates greater ACE.

For peer review only
